# Two distinct non-ribosomal peptide synthetase-independent siderophore synthetase gene clusters identified in *Armillaria* and other species in the Physalacriaceae

**DOI:** 10.1093/g3journal/jkad205

**Published:** 2023-10-16

**Authors:** Deborah L Narh Mensah, Brenda D Wingfield, Martin P A Coetzee

**Affiliations:** Departments of Biochemistry, Genetics and Microbiology, Forestry and Agricultural Biotechnology Institute (FABI), Faculty of Natural and Agricultural Sciences, University of Pretoria, Pretoria 0002, South Africa; CSIR—Food Research Institute, Microbiology and Mushroom Research Division, P. O. Box, M20, Accra, Ghana; Departments of Biochemistry, Genetics and Microbiology, Forestry and Agricultural Biotechnology Institute (FABI), Faculty of Natural and Agricultural Sciences, University of Pretoria, Pretoria 0002, South Africa; Departments of Biochemistry, Genetics and Microbiology, Forestry and Agricultural Biotechnology Institute (FABI), Faculty of Natural and Agricultural Sciences, University of Pretoria, Pretoria 0002, South Africa

**Keywords:** *Armillaria*, secondary metabolite gene clusters, ferric iron uptake, iron homeostasis, NIS synthetase, phytopathogens

## Abstract

Siderophores are important for ferric iron solubilization, sequestration, transportation, and storage, especially under iron-limiting conditions such as aerobic conditions at high pH. Siderophores are mainly produced by non-ribosomal peptide synthetase-dependent siderophore pathway, non-ribosomal peptide synthetase-independent siderophore synthetase pathway, or the hybrid non-ribosomal peptide synthetases/non-ribosomal peptide synthetases-independent siderophore pathway. Outcompeting or inhibition of plant pathogens, alteration of host defense mechanisms, and alteration of plant-fungal interactions have been associated with fungal siderophores. To understand these mechanisms in fungi, studies have been conducted on siderophore biosynthesis by ascomycetes with limited focus on the basidiomycetes. *Armillaria* includes several species that are pathogens of woody plants and trees important to agriculture, horticulture, and forestry. The aim of this study was to investigate the presence of non-ribosomal peptide synthetases-independent siderophore synthetase gene cluster(s) in genomes of *Armillaria* species using a comparative genomics approach. Iron-dependent growth and siderophore biosynthesis in strains of selected *Armillaria* spp. were also evaluated in vitro. Two distinct non-ribosomal peptide synthetases-independent siderophore synthetase gene clusters were identified in all the genomes. All non-ribosomal peptide synthetases-independent siderophore synthetase genes identified putatively encode Type A′ non-ribosomal peptide synthetases-independent siderophore synthetases, most of which have IucA_IucC and FhuF-like transporter domains at their N- and C-terminals, respectively. The effect of iron on culture growth varied among the strains studied. Bioassays using the CAS assay on selected *Armillaria* spp. revealed in vitro siderophore biosynthesis by all strains irrespective of added FeCl_3_ concentration. This study highlights some of the tools that *Armillaria* species allocate to iron homeostasis. The information generated from this study may in future aid in developing molecular based methods to control these phytopathogens.

## Introduction

All eukaryotes, and most prokaryotes, utilize iron as an essential micronutrient for various functions. However, iron is usually not bioavailable for use by these organisms under aerobic conditions at neutral pH (Chipperfield and Ratledge 2000). Hence, organisms have developed various mechanisms for ferric and ferrous iron uptake. One of the important mechanisms of ferric iron (Fe^3+^) uptake, storage, and transport used by various organisms is through synthesis and/or use of the low molecular weight (usually <1 kDa) secondary/specialized metabolites, known as siderophores (Ghosh *et al.* 2020).

Biosynthesis and secretion of siderophores occur mainly through the non-ribosomal peptide synthetase (NRPS)-dependent siderophore pathway or NRPS-independent siderophore (NIS) synthetase pathway (Kadi and Challis 2009; Brandenburger *et al.* 2017). A third pathway for siderophore biosynthesis and secretion is the hybrid NRPS/NIS pathway, which utilizes biosynthesis enzymes belonging to both of the main pathways (Lee *et al.* 2007). Genes, including the main biosynthetic genes (the backbone genes), involved in these pathways are contained in biosynthetic gene clusters (BGCs) or operons.

The main biosynthetic genes in the clusters code for enzymes that are responsible for the actual biosynthesis of the respective secondary metabolite. NRPS, the protein encoded by the backbone gene in NRPS-dependent pathway, employ discrete functional units with specific essential catalytic domains (Brandenburger *et al.* 2017). As the name suggests, NIS synthetases are the proteins encoded by the backbone biosynthetic gene of the NIS synthetase pathway. Some siderophores such as petrobactin (formerly anthrachelin) are biosynthesized via the hybrid NRPS/NIS pathway. This pathway is composed of BGCs or operons which have genes encoding both an NRPS-dependent siderophore synthetase and 1 or more NIS synthetase as the backbone genes (Lee *et al.* 2007; Nusca *et al.* 2012). Other genes in the BGCs involved in all of these pathways function in substrate modification, cluster regulation, and product transport (Dixon *et al.* 2012; Iftime *et al.* 2016; Brandenburger *et al.* 2017; Le Govic *et al.* 2018; Jarmusch *et al.* 2021).

Unlike NRPS, NIS synthetases act individually and use free intermediates as substrates (Oves-Costales *et al.* 2009). NIS synthetases are classified into 3 major superfamilies as follows: type A, B, and C NIS synthetases, based on their substrate specificity for polyamines or amino alcohols as well as the substrate activated for condensation (Oves-Costales *et al.* 2009). Type A synthetases utilize citric acid, Type B synthases utilize α-ketoglutaric acid, and type C synthases utilize monoamide/monoester derivatives of citric acid or monohydroxamate derivatives of succinic acid as their respective preferred carboxylic acid substrates (Oves-Costales *et al.* 2009). The type A NIS synthetases condense their substrates with various amines and alcohols, while the type B NIS synthetases condense their substrates with only amines. Type C NIS synthetases condense simple mono-amides or amines with a citryl or succinyl intermediate. A sub-category of the Type A NIS synthetases known as Type A′ NIS synthetases specifically catalyzes condensation of citric acid with amines (Kadi and Challis 2009). Additionally, a sub-category of the Type C NIS synthetases is known as Type C′ NIS synthetases (Carroll and Moore 2018). These enzymes mainly condense more complex citryl or succinyl intermediates but may also perform macro-cyclization to fully form a siderophore (Carroll and Moore 2018).

Genomes of some organisms contain different BGCs or operons belonging to the 2 main siderophore biosynthesis pathways (Mahe *et al.* 1995; Franza *et al.* 2005; Cheung *et al.* 2009; Cotton *et al.* 2009; Carmichael *et al.* 2019). Alternatively, the clusters could belong to 1 of the 2 main biosynthesis pathways and 1 hybrid NRPS/NIS pathway as reported in *Bacillus* spp. (Koppisch *et al.* 2005; Wilson *et al.* 2006; Oves-Costales *et al.* 2007). These BGCs or operons synthesize and transport different siderophores which have various functions such as growth promotion, growth characteristics (e.g. conidiation, dimorphism transition, and pigmented microsclerotium formation), pathogenicity, virulence, and resistance to oxidative stress (Franza *et al.* 2005; Koppisch *et al.* 2005; Li *et al.* 2016). The different siderophores are synthesized sequentially (Franza *et al.* 2005; Koppisch *et al.* 2005; Li *et al.* 2016).

Studies investigating the molecular and biochemical bases of siderophore biosynthesis, secretion, and uptake have advanced our understanding of these molecules. Minimal research that focuses their attention on the role of siderophores in fungi has been conducted (Carroll and Moore 2018). Among fungi, studies in this area have largely been focused on the Ascomycota (Haas *et al.* 2008). The first ever characterized fungal NIS synthetase, Rfs, which is involved in rhizoferrin (polyhydroxycarboxylamide siderophore) biosynthesis in the opportunistic human pathogen, *Rhizopus delemar*, was reported by Carroll *et al.* (2017). Rhizoferrin biosynthesis in *R. delemar* requires only Rfs (Carroll *et al.* 2017; Ramakrishnan *et al.* 2019). In contrast, rhizoferrin biosynthesis by the bacterial animal pathogen, *Francisella tularensis*, requires an NIS synthetase (FslA) and a pyridoxal phosphate-dependent decarboxylase (FslC) (Ramakrishnan *et al.* 2019).

Considering the importance of iron homeostasis for diverse functions in various organisms, the wide knowledge gap in siderophore biosynthesis, uptake, and utilization mechanisms in the Basidiomycota needs to be narrowed. Recently, we reported on secondary metabolite gene clusters (SMGCs) in the genomes of various *Armillaria* spp. and other members of the family Physalacriaceae, with an emphasis on NRPS-dependent siderophore synthetase gene clusters (Narh Mensah *et al.* 2023). The study revealed that the genomes investigated, except for *Cylindrobasidium torrendii*, contained SMGCs classified as siderophore gene clusters (SGCs) (Narh Mensah *et al.* 2023).

The aim of the present study was to further investigate the SGCs in the genomes of *Armillaria* spp. and other members of the Physalacriaceae. These SGCs were earlier suggested by Narh Mensah *et al.* (2023) to produce siderophores through the NIS pathway, but this was not studied further. For the purpose of the current study, the SGCs were explored using a similar comparative genomics approach to the study reported by Narh Mensah *et al.* (2023). Bioassays were also conducted to assess the iron-dependent growth and siderophore biosynthesis by strains of *Armillaria* spp. This knowledge is essential for gaining further understanding of the genomic basis of the molecular and cellular mechanisms underlying iron homeostasis in *Armillaria* spp. aimed at developing more effective control strategies of these phytopathogens in future.

## Materials and methods

### In silico identification, annotation, and characterization of NIS synthetase gene clusters

#### Cluster identification, annotation, and synteny analyses

Source information of the genomes studied are presented in Supplementary Table 1. Genome mining, genome walking, and gene annotation of selected members of the Physalacriaceae followed that described by Narh Mensah *et al.* (2023) with some modification (Steps 1–3, Supplementary Fig. 1). Cluster boundaries were predicted with Cluster Assignment by Islands of Sites (CASSIS) algorithm (Wolf *et al.* 2016) implemented in Antibiotics & Secondary Metabolite Analysis SHell (antiSMASH) using the fungal version (fungiSMASH) v 6.1.1 (Blin *et al.* 2021) in the respective genomes. These genomes were annotated in CLC Main Workbench v21.0.4 (www.qiagenbioinformatics.com) using RNA sequence data of the respective genomes (Supplementary Fig. 1). The fact that these genes are expressed therefore makes it unlikely that these genes are pseudogenes. Manual identification of NIS synthetase gene clusters, which may have been missed by fungiSMASH, was conducted by BLAST searches using CLC Main Workbench. Nucleotide sequences of NIS synthetase genes identified in the clusters of some of the genomes were used as search terms for this purpose. Gene prediction of the manually detected gene cluster was achieved with AUGUSTUS v. 3.3.3 (Stanke *et al.* 2008) (https://bioinf.uni-greifswald.de/augustus/submission.php). Flanking genes, their annotations and characteristics were determined, and gene cluster synteny maps were generated as previously described (Narh Mensah *et al.* 2023).

#### Comparison with other BGCs

Similarity of the detected SGCs to SGCs in other genomes was also investigated. For this purpose, Minimum Information about a Biosynthetic Gene cluster (MIBiG) cluster comparison (Medema *et al.* 2015; Blin *et al.* 2021), ClusterBlast (Medema *et al.* 2011), KnownClusterBlast (Blin *et al.* 2019), Cluster Pfam Analysis (Medema *et al.* 2015; Blin *et al.* 2021), and Pfam-based GO term annotation (Medema *et al.* 2015; Blin *et al.* 2021) were used. All of these bioinformatic tools are implemented in fungiSMASH (Step 4, Supplementary Fig. 1).

#### Phylogenetic analyses of NIS synthetases

A database of amino acid sequences of identified NIS synthetase genes in the genomes studied was generated with CLC Main Workbench. Additional amino acid sequences of known NIS synthetase genes available on GenBank were also added to the dataset. The final dataset (45 amino acid sequences) consisted of 19 putative NIS synthetases of *Armillaria* and other members of the Physalacriaceae, 6 orthologous proteins (selected based on high Query Cover and Percentage Identity, and low Expected value based on BLASTp searches) from GenBank, and 20 validated or proposed NIS synthetases as reported by Carroll and Moore (2018) and Oves-Costales *et al.* (2009). The sequences were used in a phylogenetic analysis to predict to which type(s) the detected NIS synthetases in the Physalacriaceae belonged (Step 5, Supplementary Fig. 1).

Multiple sequence alignments were conducted in MAFFT (Katoh *et al.* 2019), and phylogenies of the dataset were inferred using Molecular Evolutionary Genetics Analysis (MEGA) version X software (Stecher *et al.* 2020). A phylogenetic tree was inferred by using the Maximum Likelihood method. The LG + G + I substitution model was identified, by a substitution model test implemented in MEGAX, as the best substitution model based on the Bayesian Information Criterion (BIC). The initial tree(s) for the heuristic search were obtained automatically by applying Neighbor Joining (Saitou and Nei 1987) or BioNJ (Gascuel 1997) algorithms to a matrix of pairwise distances, and then selecting the topology with superior log likelihood value. A discrete Gamma distribution was used to model evolutionary rate differences among sites (5 categories [+*G*, parameter = 1.5511)]. All positions with less than 95% site coverage were eliminated, i.e. fewer than 5% alignment gaps, missing data, and ambiguous bases were allowed at any position (partial deletion option). This results in 428 character sites in the final dataset. Nodal support was determined using Bootstrap analysis with 1,000 replications (Felsenstein 1985). The tree was exported as a Newick file and visualized using FigTree version 1.4.4 (Rambaut 2018).

#### Determination of domain architecture, size, and exon number of NIS synthetase genes

Characteristics of the NIS synthetase genes (domain architectures, protein size in amino acids, and exon number) were obtained from fungiSMASH and confirmed using the InterPro web-based tool (Blum *et al.* 2021) (https://www.ebi.ac.uk/interpro/) based on results retrieved from the Pfam database (Mistry *et al.* 2021) (Step 6, Supplementary Fig. 1).

### Cultivation and assessment of siderophore biosynthesis potential of selected *Armillaria* strains

#### Growth medium preparation

To avoid iron contamination, all experiments were performed using glassware washed with HCl (6 M) and rinsed 3 times with ddH_2_0 (Schwyn and Neilands 1987; Cox 1994). Sterile plastic Petri dishes were used in the growth studies.

Strains were maintained on a malt yeast extract agar medium (15-g/L malt extract, 2-g/L yeast extract, 15-g/L agar) in 6.5-cm disposable Petri dishes. A medium without iron (PDP−) was prepared with Potato Dextrose (24 g/L) supplemented with peptone (2 g/L). A solid medium (PDPA−) was prepared with addition of agarose (10 g/L) instead of agar since agar contains iron as discussed in Giuliano Garisto Donzelli *et al.* (2015). For iron replete conditions, PDP− and PDPA− were supplemented with 100-µM FeC1_3_·6H_2_O (hereon referred to as PDP+ and PDPA+, respectively). To obtain different concentrations of added iron in PDP−, FeC1_3_·6H_2_O was added to the desired concentration. Potato dextrose was used for these experiments as it is typically used in the growth medium for Basidiomycota strains, and also to avoid very slow growth of the strains. Addition of 100-µM FeC1_3_·6H_2_O to the medium was done as it represents a relatively high concentration at which little to no siderophores are usually biosynthesized by various organisms (Alexander and Zuberer 1991; Manwar *et al.* 2004). All cultures were incubated at 25 ± 2°C in the dark.

#### Iron-dependent culture growth on solid media

Nine strains representing 6 *Armillaria* spp. (Table 1) were evaluated in this experiment. All the strains, excluding *Armillaria luteobubalina* strain CMW4977, were previously included in the study of Narh Mensah *et al.* (2023), and therefore, it is known that these strains synthesize different types of siderophores in solid media. The solid media, PDPA− and PDPA+, were used to investigate the iron-dependent culture growth rate and macromorphology of the respective strains. Each plate was inoculated with 1 5-mm diameter culture disc of actively growing culture of the respective strains. Plates were incubated for 6 weeks. Perpendicular lines intersecting at the center of the inoculum were drawn at the bottom of each Petri dish. Marks were made at the tip of the mycelia/rhizomorph in the same perpendicular angle at the end of each week to avoid disrupting incubation of the strains. Both the top and bottom views of the plates were photographed at the end of the 6-week incubation period. Radial lengths using the weekly marks were measured with the ImageJ software (https://imagej.nih.gov/ij/download.html).

**Table 1.** *Armillaria* spp. lifestyle, culture codes, source information, and CAS agar reactivity.

Species	Lifestyle^a^	CMW number	Host	Original source	CAS agar reactivity^b^
*Armillaria fuscipes*	Facultative necrotroph/pathogenic	CMW2740	*Pinus elliottii*	Entabeni, South Africa	Orange, purplish-red, purple
		CMW3164	*Pelargonium asperum*	La Réunion	Orange, purplish-red, purple
*Armillaria gallica*	Facultative necrotroph/weakly pathogenic	CMW31092	N/A	Veneto, Bellune, Italy	Reddish-orange
		CMW45397	Duff	Minnesota, USA	Reddish-orange
*Armillaria luteobubalina*	Facultative necrotroph/pathogenic	CMW4974	N/A	Australia	Orange, purplish-red
		CMW4977	N/A	Australia	ND
*Armillaria mellea*	Facultative necrotroph/highly pathogenic	CMW31132	*Ailanthus altissima*	China	Orange, reddish-orange, purplish-red, purple
*Armillaria nabsnona*	Weakly pathogenic	CMW3159	*Acer macrophyllum*	Vancouver, Canada	Reddish-orange, purplish-red, purple
		CMW6904	*Acer circinatum*	USA	Orange, purplish-red
*Armillaria* sp. ACB	N/A	CMW4456	*Brachystegia utilis*	Stapleford, Zimbabwe	Orange, purple

Modified from Narh Mensah *et al.* (2023).

ACB, African Clade B in Coetzee *et al*. (2005); N/A, not available; ND, not determined.

^a^Lifestyle information are summarized from Koch *et al.* (2017).

^b^Color change as determined by universal CAS and modified CAS agar assays based on images in Narh Mensah *et al.* (2023).

#### Evaluation of iron-dependent siderophore biosynthesis

Firstly, siderophore biosynthesis under iron deplete and iron replete conditions was evaluated for all the strains of *Armillaria* species included in this study (Table 1). The respective cultures (1-cm^2^ culture disks) were aseptically inoculated into 50-mL falcon tubes containing 15-mL PDP− or PDP+. The cultures were incubated for 24 hours. Secondly, the iron-dependent repressibility of siderophore biosynthesis was also investigated. For this purpose, 1-cm^2^ culture disk of *A. fuscipes* strain CMW2740 and *A. mellea* strain CMW31132 was separately inoculated in 15-mL PDP amended with FeCl_3_ at different concentrations (0, 20, 40, 60, 80, 100, 150, 200 µM) in 50-mL falcon tubes and incubated for 24 hours.

For both experiments, siderophore biosynthesis was detected using the modified CAS assay solution with the microtiter method according to Alexander and Zuberer (1991). Cultures were swirled to mix, and aliquots (100 µL) of supernatants were mixed with 100 µL of CAS assay solution in 96-well plates (TPP Tissue Culture Test Plate 96F, Switzerland). Absorbance readings were done in a SpectraMax Plus 384 Spectrophotometer (Labotec, South Africa) at 630 nm after 20 minutes. Siderophore production was quantified in percentage siderophore units (psu) using the formula as follows:


$$\mathrm{Siderophore}\;\mathrm{production}\;(\mathrm{psu})\;=\;\frac{(A_{r}-A_{s})\times 100}{A_{r}}$$

where *A*_r_ = absorbance of reference [CAS solution and un-inoculated broth (control)], and *A*_s_ = absorbance of sample (CAS solution and cell-free supernatant of sample) (Payne 1994).

#### Statistical analysis

Growth experiments were performed in biological triplicates. For iron-dependent siderophore biosynthesis and iron-repressibility of siderophore biosynthesis experiments, we repeated both experiments twice independently and with 3 biological replicates per treatment. All data were analyzed using Microsoft Excel. The mean and standard errors of all reads for the growth experiment were determined at the fourth week of incubation. This was reported as the radial culture length at week 4. Statistical significance of the radial culture lengths between treatments for each strain was evaluated using *t*-test. One-tailed paired 2-sample for means were calculated for comparison of all strains studied excluding strains CMW3159 and CMW4456. Due to the irregular and rhizomorphic growth patterns of these 2 strains, the 1-tailed 2-sample assuming equal variances *t*-test was performed for CMW3159 and CMW4456. A 95% probability level was used in all analyses. Growth rates were calculated by the linear regression obtained by the fourth week of incubation. Iron-repressibility of siderophore biosynthesis was means and standard errors for each concentration of iron.

## Results

### Comparative genomics analyses

#### Identified NIS synthetase gene clusters and cluster annotation

**Fig. 1.**
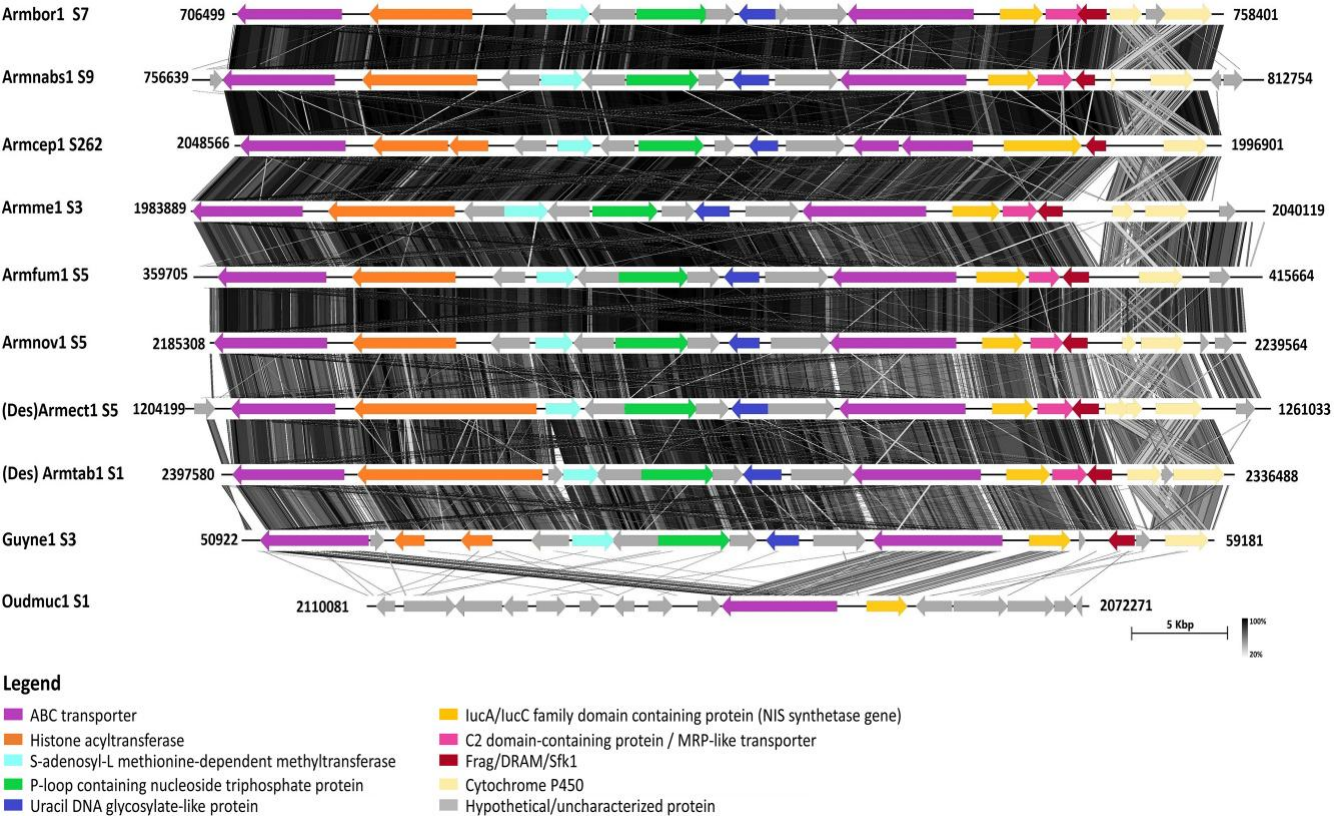
Synteny map of NIS Cluster 1 and neighboring genes in annotated genomes of Physalacriaceae species. From top to bottom, NIS Cluster 1 in genomes of *A. borealis* (Armbor1), *A. nabsnona* (Armnabs1), *A. cepistipes* (Armcep1), *A. mellea* (Armme1), *A. fumosa* (Armfum1), *A. novae-zelandiae* (Armnov1), *Desarmillaria ectypa* [(Des)Armect1], *Desarmillaria tabescens* [(Des)Armtab1], *Guyanagaster necrorhizus* (Guyne1), and *Oudemansiella mucida* (Oudmuc1) is presented. Numbers following the species code are the scaffolds (S) on which the clusters are located. Numbers at the ends of the clusters are the locations on the scaffolds. Different colors (different putative proteins as determined by tBLASTn searches) and orientation of arrows (direction of transcription) are shown. Orthologous genes are identically colored. For Oudmuc1 S1, only orthologous genes are identically colored. Darker shades of lines between clusters represent higher amino acid similarity between the respective clusters based on tBLASTx.

**Fig. 2.**
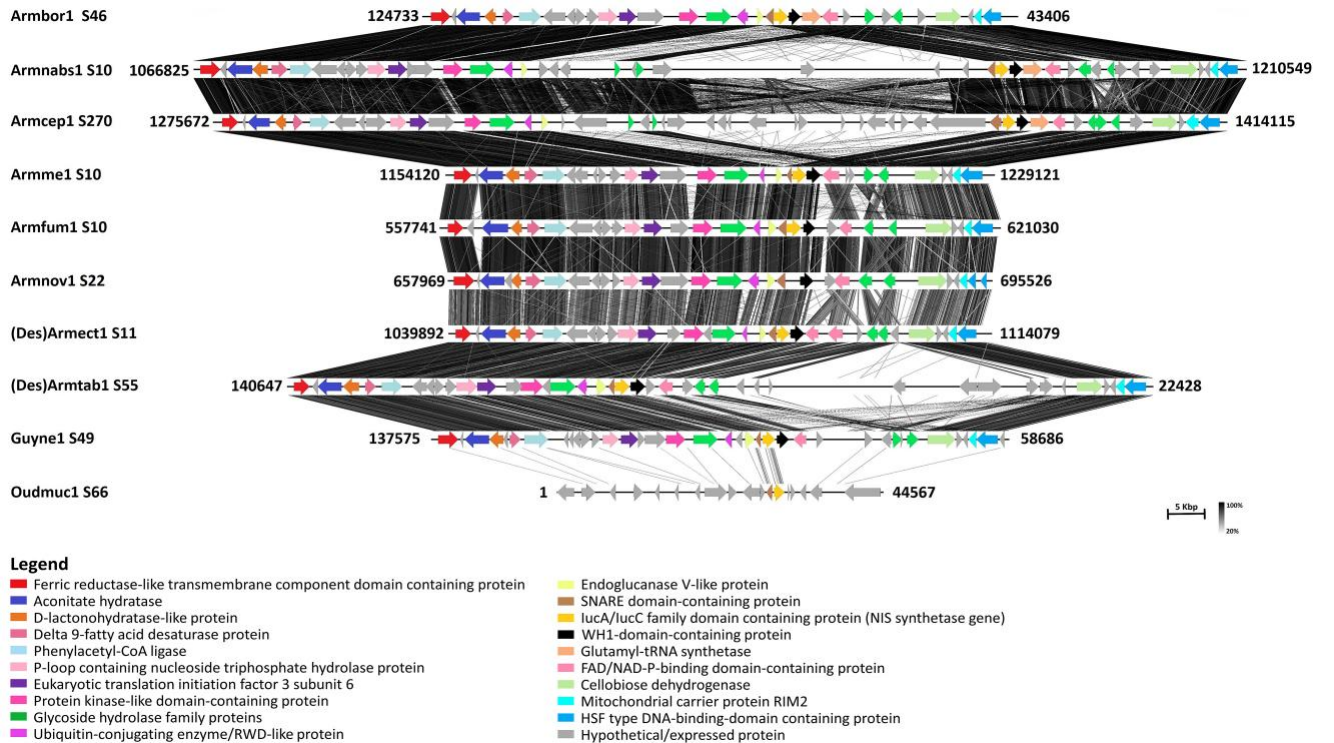
Synteny map of NIS Cluster 2 and neighboring genes in annotated genomes of Physalacriaceae species. From top to bottom, NIS Cluster 2 in genomes of *A. borealis* (Armbor1), *A. nabsnona* (Armnabs1), *A. cepistipes* (Armcep1), *A. mellea* (Armme1), *A. fumosa* (Armfum1), *A. novae-zelandiae* (Armnov1), *D. ectypa* [(Des)Armect1], *D. tabescens* [(Des)Armtab1], *G. necrorhizus* (Guyne1), and *O. mucida* (Oudmuc1) is presented. Numbers following the species code are the scaffolds (S) on which the clusters are located. Numbers at the ends of the clusters are the locations on the scaffolds. Different colors (different putative proteins as determined by tBLASTn searches) and orientation of arrows (direction of transcription) are shown. Orthologous genes are identically colored. Only the orthologous gene is identically colored for Oudmuc1 S66. Darker shades of lines between clusters represent higher amino acid similarity between the respective clusters based on tBLASTx.

Two different NIS synthetase gene clusters (NIS Cluster 1 and NIS Cluster 2) were found in the genomes studied, excluding the genome of *C. torrendii*. These BGCs showed conserved microsynteny with retention of gene content, order, and orientation. Some gene loss and/or duplication events were, however, observed (Figs. 1 and 2).

NIS Cluster 1 (Fig. 1, Supplementary Table 2) included genes which putatively encode ATP-binding cassette (ABC) transporters, S-adenosyl-L methionine-dependent methyltransferase, iron uptake chelate (Iuc)A/IucC family domain-containing protein (NIS synthetase gene), C2 domain-containing protein/Multi-Drug Resistance Protein (MRP)-like transporter, Frag/DRAM/Sfk1, and cytochrome P450. The putative ABC transporter genes found in all the genomes studied were duplicated. The cytochrome P450 genes in NIS Cluster 1 were duplicated in some of the *Armillaria* and *Desarmillaria* gene clusters [Armbor1 S7, (Des)Armtab1 S1, and (Des)Armect1 S5]. There appeared to be remnants of the duplicated cytochrome P450 in other gene clusters of some *Armillaria* spp. and *Guyanagaster necrorhizus* (Armnabs1 S9, Armme1 S3, Armnov1 S5, and Guyne1 S3), while Oudmuc1 S1 lacked this gene.

Unlike NIS Cluster 1, NIS Cluster 2 and neighboring genes included genes which putatively encode a ferric reductase-like transmembrane component domain-containing protein, glycoside hydrolase family 61 and 95 proteins, ubiquitin-conjugating enzyme/RWD-like protein, endoglucanase V-like protein, soluble *N*-ethylmaleimide-sensitive factor attachment protein receptor (SNARE) domain-containing protein, IucA/IucC family domain-containing protein (NIS, biosynthetic backbone gene), WH1-domain-containing protein, glutamyl-tRNA synthetase, and Heat shock factor (HSF) type DNA-binding domain-containing protein (Fig. 2, Supplementary Table 2). All the NIS synthetase gene clusters contained hypothetical/expressed proteins (Figs. 1 and 2). The expected location of the second NIS synthetase gene of *Armillaria novae-zelandiae* had high similarity (>70%) with the NIS synthetase genes in *Armillaria fumosa* (Armfum1 S10) and *Desarmillaria ectypa* [(Des)Armect1 S11] (Fig. 2).

#### Similarity of CASSIS-determined cluster boundaries to other BGCs

The cluster boundaries of NIS Cluster 1 as predicted by the CASSIS algorithm included the genes encoding IucA/IucC family domain-containing protein (NIS synthetase, biosynthetic backbone gene), C2 domain-containing protein/MRP-like transporter, and Frag/DRAM/Sfk1 in the genomes of most of the *Armillaria* and *Desarmillaria* spp. (Table 2; Supplementary File 1). NIS Cluster 1 of *Armillaria nabsnona* (Armnabs1 S9) and *D. ectypa* [(Des)Armect1 S5] also contained a gene encoding cytochrome P450, whereas that of *Armillaria cepistipes* (Armcep1 S262) lacked the gene encoding C2 domain-containing protein/MRP-like transporter. NIS Cluster 1 in the genome of *G. necrorhizus* (Guyne1 S3) appeared to have a remnant of the gene encoding C2 domain-containing protein/MRP-like transporter. This gene has putatively been annotated as a hypothetical protein in Guyne1 S3. The cluster boundary for NIS Cluster 1 of *Oudemansiella mucida* (Oudmuc1 S1) included genes which encode IucA/IucC family domain-containing protein, WD repeat-containing protein 8, and Major Facilitator Superfamily (MSF) transporter (Table 2).

**Table 2.** CASSIS-determined NIS synthetase gene cluster boundaries, MIBiG comparison and ClusterBlast hits.

	NIS Cluster 1			NIS Cluster 2		
Genome^a^	CASSIS gene cluster^b^	MIBiG comparison	ClusterBlast hit^c^	CASSIS gene cluster^b^	MIBiG comparison^d^	ClusterBlast hit
*A. borealis* (Armbor1 S7 and S46)	*IucA*/*IucC*, C2 domain-containing protein/MRP-like transporter, Frag/DRAM/Sfk1	ND	NW_006267366 (1141250–1156888)	Ubiquitin-conjugating enzyme/RWD-like protein, Endoglucanase V-like protein, SNARE domain-containing protein, *IucA*/*IucC*, WH1-domain-containing protein, and Glutamyl-tRNA synthetase	BGC0000944.1, 0.13, staphyloferrin A	ND
*A. nabsnona* (Armnabs1 S9 and S22)	*IucA*/*IucC*, C2 domain-containing protein/MRP-like transporter, Frag/DRAM/Sfk1, Cytochrome P450	ND	NW_006267366 (1141250–1156888)	Hypothetical protein, SNARE domain-containing protein, *IucA*/*IucC*, WH1-domain-containing protein, and Glutamyl-tRNA synthetase	BGC0000944.1, 0.13, staphyloferrin A	ND
*A. cepistipes* (Armcep1 S262 and S270)	*IucA*/*IucC*, Frag/DRAM/Sfk1	ND	NW_006267366 (1141250–1156888)	SNARE domain-containing protein, *IucA*/*IucC*, WH1-domain-containing protein, and Glutamyl-tRNA synthetase	ND	ND
*A. mellea* (Armme1 S3 and S10)	*IucA*/*IucC*, C2 domain-containing protein/MRP-like transporter, Frag/DRAM/Sfk1	ND	NW_006267366 (1141250–1156888)	Ubiquitin-conjugating enzyme/RWD-like protein, Endoglucanase V-like protein, SNARE domain-containing protein, *IucA*/*IucC*, WH1-domain-containing protein, and FAD/NAD-P-binding domain-containing protein	BGC0000944.1, 0.13, staphyloferrin A	ND
*A. fumosa* (Armfum1 S5 and S10)	*IucA*/*IucC*, C2 domain-containing protein/MRP-like transporter, Frag/DRAM/Sfk1	ND	NW_006267366 (1141250–1156888)	Ubiquitin-conjugating enzyme/RWD-like protein, Endoglucanase V-like protein, SNARE domain-containing protein, *IucA*/*IucC*, and WH1-domain-containing protein	BGC0000944.1, 0.13, staphyloferrin A	ND
*A. novae-zelandiae* (Armnov1 S5 and S22)	*IucA*/*IucC*, C2 domain-containing protein/MRP-like transporter, Frag/DRAM/Sfk1	ND	NW_006267366 (1141250–1156888)	N/A	N/A	N/A
*D. ectypa* [(Des)Armect1 S5 and S11]	*IucA*/*IucC*, C2 domain-containing protein/MRP-like transporter, Frag/DRAM/Sfk1, Cytochrome P450	ND	NW_006267366 (1141250–1156888)	Ubiquitin-conjugating enzyme/RWD-like protein, Endoglucanase V-like protein, SNARE domain-containing protein, *IucA*/*IucC*, WH1-domain-containing protein, and FAD/NAD-P-binding domain-containing protein	BGC0000944.1, 0.13, staphyloferrin A	ND
*D. tabescens* [(Des)Armtab1 S1 and S55]	*IucA*/*IucC*, C2 domain-containing protein/MRP-like transporter, Frag/DRAM/Sfk1	ND*	NW_006267366 (1141250–1156888)	Endoglucanase V-like protein, SNARE domain-containing protein, *IucA*/*IucC*, WH1-domain-containing protein, and FAD/NAD-P-binding domain-containing protein related to salicylate hydroxylase	BGC0000944.1, 0.13, staphyloferrin A	ND
*G. necrorhizus* (Guyne1S3 and S49)	*IucA*/*IucC*, C2 domain-containing protein/MRP-like transporter, Frag/DRAM/Sfk1, hypothetical protein	ND	NW_006267366 (1141250–1156888)	Ubiquitin-conjugating enzyme/RWD-like protein, Endoglucanase V-like protein, SNARE domain-containing protein, *IucA*/*IucC*, WH1-domain-containing protein, and FAD/NAD-P-binding domain-containing protein	BGC0000944.1, 0.13, staphyloferrin A	ND
*O. mucida* (Oudmuc1 S1 and S66)	*IucA*/*IucC*, WD repeat-containing protein 8, MFS general substrate transporter	ND*	ND	small nucleolar RNA-associated protein 3, ribosomal protein S12, SNARE domain-containing protein, *IucA*/*IucC*, and 3 hypothetical proteins	BGC0000944.1, 0.13, staphyloferrin A	ND

ND, none detected; ND*, all hits had no similarity to the biosynthetic backbone gene, *IucA*/*IucC*; N/A, not applicable (cluster detected by BLAST search in CLC).

^a^Presented as species [genome code and scaffold (S) numbers on which NIS Clusters 1 and 2 are located, respectively].

^b^Genes in cluster boundary detected by CASSIS.

^c^100% of genes in the hit show similarity to the genes in the query sequence (i.e. the sequence of NIS synthetase gene clusters of the Physalacriaceae).

^d^Presented as reference of hit in MIBiG database, similarity score, compound synthesized by the hit.

Genes encoding SNARE domain-containing protein, IucA/IucC family domain-containing protein, WH1-domain-containing protein, and glutamyl-tRNA synthetase were predicted as the boundaries of NIS Cluster 2 by CASSIS algorithm in *Armillaria borealis* (Armbor1 S46), *A. nabsnona* (Armnabs1 S10), and *A. cepistipes* (Armcep1 S270). The genes in the cluster boundaries of all NIS Cluster 2 in the other studied genomes, except that of *O. mucida* (Oudmuc1 S66), encode all the indicated proteins excluding glutamyl-tRNA synthetase. The cluster boundary for Oudmuc1 S66 consisted of genes which encode small nucleolar RNA-associated protein 3, ribosomal protein S12, SNARE domain-containing protein, IucA/IucC, and 3 hypothetical proteins (Table 2; Supplementary File 1).

No MIBiG comparison hit was recorded for NIS Cluster 1 of all the studied genomes apart from NIS Cluster 1 in the genomes of *D. tabescens* [(Des)Armtab1 S1] and *O. mucida* (Oudmuc1 S1), in which there were hits with no similarity to the biosynthetic backbone gene, IucA/IucC (Table 2). MIBiG comparison of NIS Cluster 2 in the studied genomes revealed that a region, BGC0000944.1 (location, 2257888–2263981), responsible for staphyloferrin A biosynthesis in *Staphylococcus aureus* subsp. *aureus* NCTC 8325 (Cotton *et al.* 2009) had a similarity score of 0.13 in the MIBiG database (Table 2). This excluded NIS Cluster 2 of *A. cepistipes* (Armcep1 S270).

Results from ClusterBlast showed NIS Cluster 1 in all the genomes excluding that of Oudmuc1 S1 had 100% gene similarity (in terms of the proteins encoded by the genes) within the BGC of *Agaricus bisporus* var. *bisporus* H97, (NW_006267366; location, 1141250–1156888) (Table 2). No similar region was recorded for NIS Cluster 2 in all the genomes. BLASTp hits of the genes in NIS Cluster 2 of *A. borealis* to this region are shown in Supplementary Table 3. No KnownClusterBlast hits were recorded for all the BGCs identified in this study.

#### Phylogeny of putative NIS synthetase genes

**Fig. 3.**
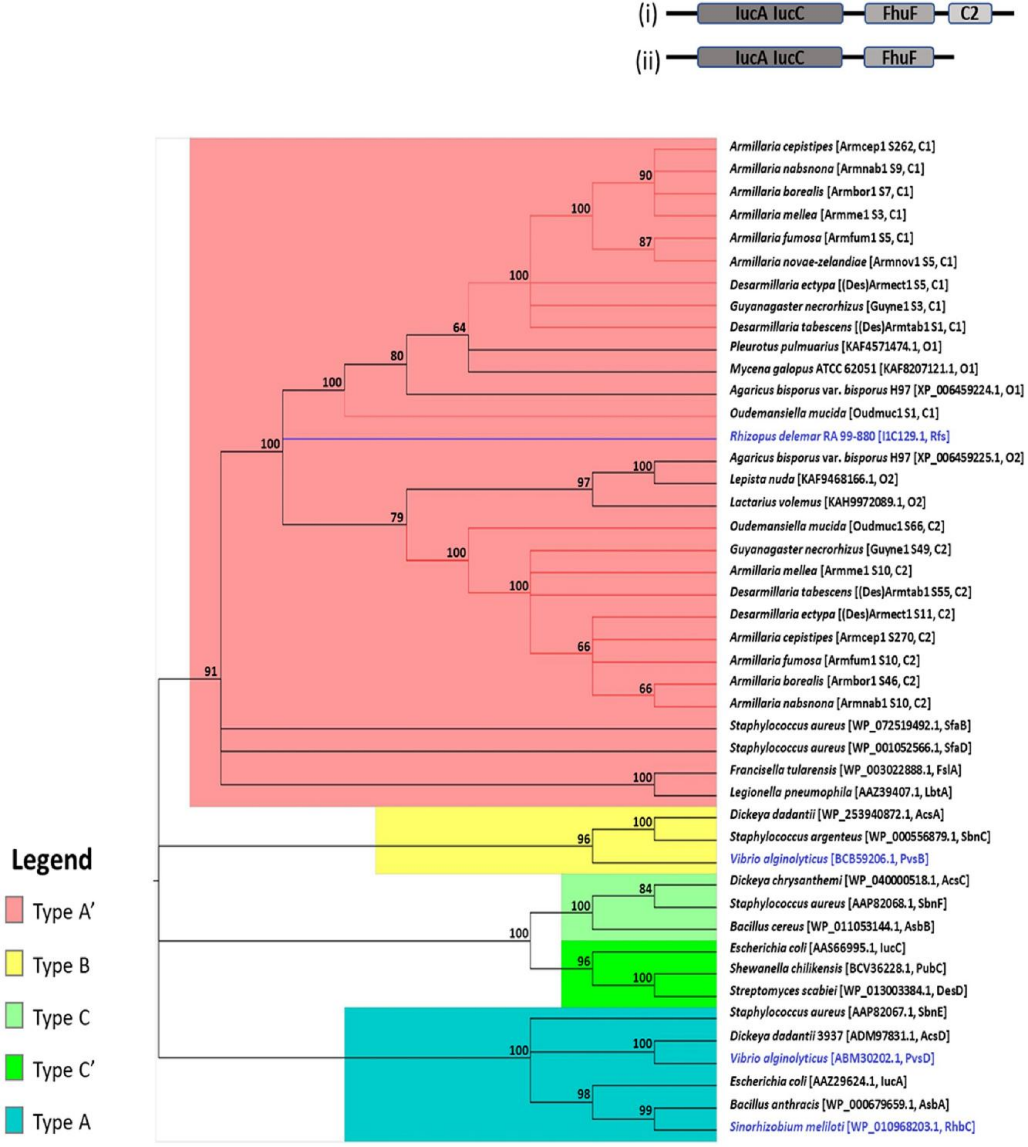
Cladogram showing the NIS synthetase genes in both NIS Clusters 1 and 2 grouping with known Type A′ NIS synthetases and some uncharacterized orthologs in the Basidiomycota. Branches colored rose show NIS synthetases belonging to the Physalacriaceae. Labels of NIS synthetases in NIS Clusters 1 and 2 are presented as species [genome code scaffold number on which the cluster was located in the genome, cluster number]. C1 and C2 represent NIS Cluster 1 and NIS Cluster 2, respectively. The other NIS synthetases used in the analyses are labeled as species name [accession number, name of NIS synthetase]. O1 and O2are orthologs obtained from BLASTp searches with protein sequences of the NIS synthetases in NIS Clusters 1 and 2, respectively, as well as the BLASTp hit of the similar cluster in *Agaricus bisporus* var. *bisporus* H97 obtained from fungiSMASH. The only extensively characterized fungal NIS synthetase, Rfs, is indicated in blue text. Other protein names are indicated in black and blue text for characterized and uncharacterized NIS synthetases used in the dataset, respectively. Bootstrap values greater than 60% are shown next to the nodes. Inserts at the top right corner are the Pfam domain architectures of all the NIS synthetases. Insert (i) is Pfam domain of the NIS synthetase of Armcep1 S262 (Armcep1_13598) showing the IucA_IucC, ferric iron reductase FhuF-like transporter, and C2 domains. Insert (ii) is Pfam domain architectures of all other NIS synthetases in the cladogram, showing the IucA_IucC and ferric iron reductase FhuF-like transporter domains at the N- and C-terminals, respectively.

The phylogenetic analysis revealed that all the NIS synthetase genes (*IucA*/*IucC* genes) in both NIS Clusters 1 and 2 identified in the genomes studied encode Type A′ NIS synthetases although the NIS synthetases of *O. mucida* branch separately from those of the other members of the Physalacriaceae in the respective gene clusters (Fig. 3; Supplementary Table 4). Nevertheless, the NIS synthetases from the respective gene clusters formed 2 distinct highly supported phylogenetic clades (Fig. 3; 100% bootstrap value). The NIS synthetases in NIS Cluster 1 of the Physalacriaceae (C1, Fig. 3) grouped with some uncharacterized orthologs in the Basidiomycota (O1, Fig. 3; Supplementary Table 4) and with the only characterized fungal NIS synthetase from *R. delemar* RA 99–880 (Rfs, Fig. 3; Supplementary Table 4).

#### Domain architectures and other characteristics of putative NIS synthetases

Some characteristics of the putative NIS synthetases of the Physalacriaceae in comparison with NIS synthetases of other organisms are described in this section. The domain architectures of the putative NIS synthetases were similar in almost all the genomes studied and were comparable to the domain architectures of the known NIS synthetase genes included in the analysis. The NIS synthetase gene of *A. cepistipes* S262 (Armcep1_13598) contained IucA_IucC (PF04183.14) domain at the N-terminal, ferric iron reductase (Fhuf; PF06276.14) domain in the middle, and C2 domain at the C-terminal [insert (i), Fig. 3]. The NIS synthetases in all the other *Armillaria* spp. as well as the *Desarmillaria* spp. and *O. mucida* had IucA_IucC and Fhuf domains at the N- and C-terminals, respectively [insert (ii), Fig. 3]. This architecture has also been recorded in the NIS synthetases of *R. delemar* RA 99–880, Rfs (Carroll *et al.* 2017) and the uncharacterized Type A′ NIS synthetases of all the orthologs which were identified. All the other known NIS synthetases included in the phylogenetic analyses in Section 3.1.3 also had this domain architecture.

The NIS synthetases differed in size and number of exons. The sizes of the NIS synthetases from NIS Cluster 1 were 497–1132 amino acids (Supplementary Table 4) with 6–10 exons (data not shown). NIS synthetase of NIS Cluster 2 was 483–559 amino acids (Supplementary Table 4) with 1–3 exons (data not shown).

Gene prediction of the NIS synthetase gene in the manually detected NIS Cluster 2 of *A. novae-zelandiae* with AUGUSTUS v. 3.3.3 identified 2 copies. The predicted genes were 510 and 515 amino acids with 2 or 3 exons, respectively. Both genes were orthologous to IucC domain family containing proteins of *Mucidula mucida* and *Gymnopilus junonius* (accession numbers KAF8906867.1 and KAF8899909.1) with query covers and percentage identities greater than 50%. Both predictions also had the IucA_IucC and Fhuf domains at the N- and C-terminals, respectively, based on InterPro predictions (results not shown).

### Iron-dependent growth and siderophore biosynthesis

In vitro experiments were conducted to investigate the effect of iron on growth and siderophore biosynthesis by *Armillaria* species to gain some understanding of why members of this genus dedicate several genetic tools to siderophore biosynthesis and transport.

#### Iron-dependent growth

**Fig. 4.**
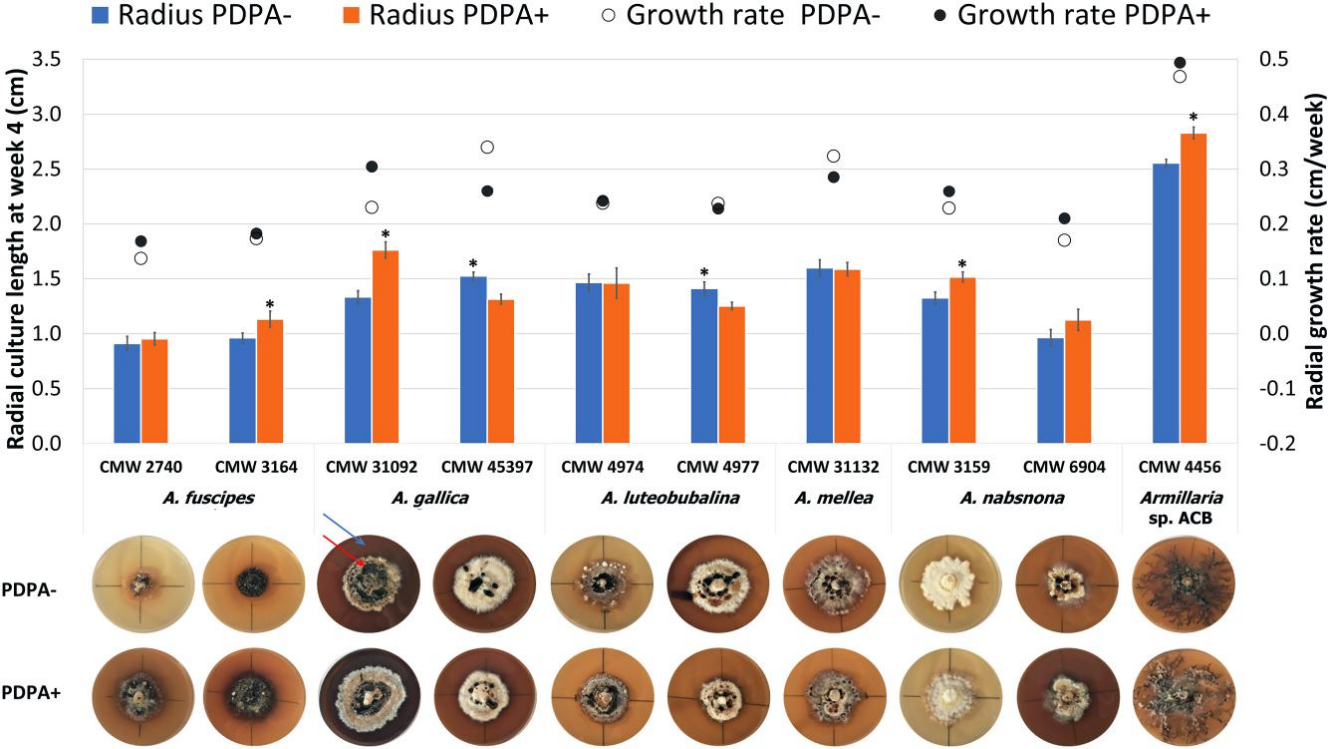
Iron-dependent mycelia growth on solid media. PDPA+ and PDPA− are Potato Dextrose Peptone Agarose with and without added 100-µM FeCl_3_, respectively. Means of radial culture lengths at week 4 are presented (n = 3). Culture lengths were taken at week 4 of incubation to avoid obtaining unrepresentative, skewed results as some of the strains fully colonized the plates at the fourth week. Error bars are standard errors of the means. Error bars marked with asterisks are significantly different (*P* < 0.05) between treatments for the respective strains. Representative plates of strains grown on PDPA− (top row) and PDPA+ (bottom row) at end of incubation showing culture macromorphology and growth, as well as secretion of brownish exudates diffused into the media (blue arrow) or as liquid on cultures (red arrow), are also presented. Plates in each column correspond to the respective strain of *Armillaria* spp.

Culture radial growth and growth rates were calculated at week 4 of incubation, and culture macromorphology by the end of incubation (week 6 of incubation) differed among the different strains studied (Fig. 4; Supplementary File 2). The lowest culture radii and growth rates were recorded for *A. fuscipes* strain CMW2740. The highest radii and growth rates were recorded for *Armillaria* spp. from African Clade B (CMW4456) (Fig. 4; Supplementary File 2). For both strains, the cultures grown on PDPA+ had a significantly longer radius than the cultures grown on PDPA− (*P* < 0.05). Between the *A. gallica* strains studied, cultures of CMW31092 showed significantly lower radii and growth rates when cultured on PDPA− than on PDPA+ (*P* < 0.05; Fig. 4). The reverse was true for *A. gallica* strain CMW45397. *R*^2^ values recorded for the linear regression obtained for all the strains were greater than 0.9 (results not shown), indicating a linear growth of all strains under the experimental conditions.

Culture macromorphology was generally similar for the strains on the respective media (Fig. 4). *A. nabsnona* strain CMW3159, however, showed a denser culture macromorphology when cultured on PDPA− compared to PDPA+ (Fig. 4). On PDPA+ cultures of *Armillaria* spp. from African Clade B (CMW4456) showed whitish aerial mycelia on the rhizomorphs. All strains secreted brownish exudates which diffused into the media and/or present as liquid on the cultures on both media (Fig. 4). Exudate secretion occurred at different degrees among the strains as shown by the change of the yellowish growth media to varying shades of brown and the presence of brown liquid on the cultures. Exudate secretion was generally more pronounced on PDPA+ as exemplified by *A. fuscipes* strains CMW2740 and CMW3164.

#### Iron-dependent siderophore biosynthesis

**Fig. 5.**
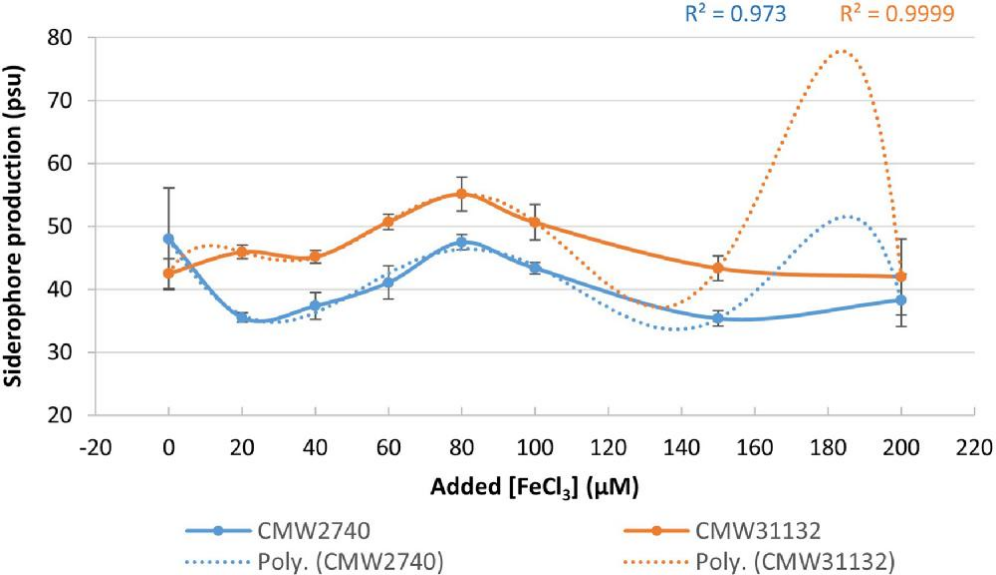
Iron-repressive siderophore biosynthesis by *A. fuscipes* strain CMW2740 and *A. mellea* strain CMW31132. Values presented are means of biological replicates. Error bars are standard errors of the means. *R*^2^ values for CMW2740 (blue) and CMW31132 (brown) are indicated. Poly. = polynomial regression with orders of 6.

All strains studied produced siderophores both under iron deplete (PDP−) and iron replete (PDP+) conditions (data not shown). The iron-repressibility of siderophore biosynthesis fluctuated for both strains. The best fits for the curves obtained followed polynomial trendlines with orders of 6 for both *A. fuscipes* strain CMW2740 (*R*^2^ = 0.973) and *A. mellea* strain CMW31132 (*R*^2^ = 0.9999) (Fig. 5; Supplementary File 3). Strain CMW31132 generally produced more siderophores than strain CMW2740 at the same concentration of added FeCl_3_ (Fig. 5; Supplementary File 3).

## Discussion

### Genome analyses reveal 2 distinct NIS synthetase gene clusters and NIS synthetase genes in the Physalacriaceae

#### NIS synthetase gene clusters in the Physalacriaceae

The presence of SGC in all the genomes studied, excluding that of *C. torrendii*, is congruent with the report of Narh Mensah *et al.* (2023). NIS synthetase gene clusters contain gene(s) which encode 1 or more types of NIS synthetase (Bull *et al.* 1996; Lynch *et al.* 2001; Moon *et al.* 2004; Deng *et al.* 2006; Cheung *et al.* 2009; Carroll *et al.* 2017). These clusters may also contain other genes involved in biosynthesis, transport, and regulation (Bull *et al.* 1996; Lynch *et al.* 2001; Moon *et al.* 2004; Deng *et al.* 2006; Cheung *et al.* 2009; Carroll *et al.* 2017). The presence of only the NIS-type backbone gene (IucA/IucC) in all the identified gene clusters in this study showed that they are NIS synthetase gene clusters and not hybrid NRPS/NIS gene clusters.

Results from this study provide some evidence that the products of the identified gene clusters could be essential for iron homeostasis in species within the investigated genera. This assertion is based on the largely conserved microsynteny in the genes and intergenic regions of both NIS Clusters 1 and 2 and their neighboring genes in the genomes of *Armillaria*, *Desarmillaria*, and *Guyanagaster* despite the observed putative gene loss and/or duplication events. Species from the same and/or different genera have previously been reported to show conserved synteny as well as some gene loss and/or duplication or other forms of gene modification events in BGCs (Goering *et al.* 2016; Tralamazza *et al.* 2019; Evdokias *et al.* 2021). Gene recruitment, duplication, repurposing, and other modification events in BGCs have been shown to impact secondary metabolism. This effect occurs through loss of gene (cluster) function or diversification of structures of the associated secondary metabolites as exemplified by ergot alkaloids biosynthesis by members of the fungal family, Clavicipitaceae (Florea *et al.* 2017). Additionally, lifestyle-independent and species-independent differential siderophore biosynthesis in vitro has been reported in *Armillaria* (Narh Mensah *et al.* 2023). Hence, the biological implications of the presently recorded putative NIS synthetase gene cluster modifications in the Physalacriaceae need to be investigated in future.

Various organisms have been reported to employ different tools for siderophore biosynthesis. The present study has shown that in addition to the previously reported NRPS-dependent siderophore synthetase gene cluster in genomes of *Armillaria* and other species within the Physalacriaceae (Narh Mensah *et al.* 2023), the genomes of these fungi also contain 2 distinct NIS synthetase gene clusters. The ability to differentially regulate and synthesize different siderophores with different biological functions using 2 of the 3 siderophore biosynthesis pathways has been reported in *Erwinia chrysanthemi* (Franza *et al.* 2005) and some other bacteria (Koppisch *et al.* 2005; Cheung *et al.* 2009; Cotton *et al.* 2009). We predict that the 2 distinct NIS synthetase gene clusters in the genomes of the respective Physalacriaceae will be regulated differently and will synthesize different siderophores which may be involved in different or overlapping functions under different conditions. This is based on the observed differences in both the NIS synthetase genes and the flanking genes in the 2 gene clusters within each of the studied genomes. Genes such as HSF type DNA-binding domain-containing protein in NIS Cluster 2 remain latent and are activated under stress conditions and in response to developmental signals to induce transcription of heat shock genes (reviewed in Pirkkala *et al.* (2001)). Heat shock proteins have been reported to be highly abundantly expressed by organisms in response to high metal concentrations (Dias *et al.* 2019; Khatiwada *et al.* 2020; Okay *et al.* 2020).

The genomes of organisms other than fungi have been shown to contain more than 1 NIS synthetase gene cluster or operon with 1 or more type(s) of NIS synthetase gene(s) in each cluster (Franza *et al.* 2005; Cotton *et al.* 2009; Carmichael *et al.* 2019). To the best of our knowledge, this is the first report of different NIS synthetase gene clusters in fungal genomes.

#### Cluster boundaries and similarities to known NIS synthetase gene clusters

Genes in the cluster boundaries of both NIS Clusters 1 and 2 of the Physalacriaceae predicted by CASSIS are typical of NIS synthetase gene clusters. These genes encode the NIS biosynthesis backbone gene (*IucA*/*IucC* in both NIS Clusters 1 and 2) and the transporter (C2 domain-containing protein/MRP-like transporter in NIS Cluster 1). Other proteins, such as ABC transporters, Cytochrome P450, Frag/DRAM/Sfk1, SNARE domain-containing protein, and Glutamyl-tRNA synthetase, encoded by genes identified in NIS Clusters 1 and 2 in the genomes of species of *Armillaria*, *Desarmillaria*, and *Guyanagaster* during this study, may function in substrate/product modification, transport, and regulation. This is supported by previous studies that showed that petrobactin acquisition in *Bacillus anthracis* is facilitated by multiple ABC transporters (Dixon *et al.* 2012). Furthermore, different authors showed that NIS synthetase gene clusters for desferrioxamine biosynthesis by *Streptomyces* spp. contain genes which encode cytochrome P450 monooxygenases (Iftime *et al.* 2016; Jarmusch *et al.* 2021). Additionally, the ferric reductase-like transmembrane component domain-containing protein gene found in NIS Cluster 2 in all the studied genomes, excluding that of *O. mucida*, may be involved in iron homeostasis in these fungi. The participation of ferric reductases in iron homeostasis has been documented in other organisms including bacteria and some fungal pathogens (Cain and Smith 2021). The putative modification of the gene encoding C2 domain-containing protein/MRP-like transporter in NIS Cluster 1 in the genome of *G. necrorhizus* and its biological implications needs to be investigated.

The gene clusters identified for *O. mucida* in this study differed from NIS Clusters 1 and 2 present in the other species. The gene clusters for *O. mucida*, however, contained genes which encode the NIS synthetase (*IucA*/*IucC* in both gene clusters) and other genes putatively involved in regulation and/or transport. Genes which encode transporters and regulators have also been reported in various NIS synthetase gene clusters of other organisms (Moon *et al.* 2004; Cheung *et al.* 2009; Iftime *et al.* 2016).

The 2 NIS synthetase gene clusters detected in the studied genomes code for proteins which are likely to biosynthesize different siderophores by the respective species. This is evidenced by the fact that the genes located in the cluster boundaries of both gene clusters in the studied genomes are different. In addition, there is no significant similarity score in the MIBiG database for NIS Cluster 1 as opposed to NIS Cluster 2 and vice versa for the ClusterBlast hits. Results from this study suggest that the product of NIS Cluster 1 in the genomes of *Armillaria*, *Desarmillaria*, and *Guyanagaster* will be similar to the product which may be synthesized by the NW_006267366 BGC of *A. bisporus* var. *bisporus* H97. Conversely, based on MIBiG comparison, the product expressed by the NIS synthetase gene of NIS Cluster 2 of all the studied genomes, excluding that of *A. cepistipes* (Armcep1 S270), will be similar to the product expressed by the gene in the BGC, BGC0000944.1: 0–6093, although the similarity score is low. This cluster synthesizes staphyloferrin A in *S. aureus* subsp. *aureus* NCTC 8325. The presence of a set of identical genes in a siderophore BGC in the genome of an organism does not necessarily translate to biosynthesis of the same siderophore as shown in putrebactin and alcaligin BGCs in *Shewanella* spp. and *Bordetella* spp., respectively (Kadi *et al.* 2008). This fact, as well as the low similarity scores presently recorded for the MIBiG comparisons, and the fact that there were no KnownClusterBlast hits for NIS Clusters 1 and 2 in all the studied genomes suggest that the identified NIS synthetase gene clusters will synthesize novel siderophores.

#### Phylogenetic analysis of putative NIS synthetases reveal that the genes in both gene clusters are Type A′ NIS synthetases

All the putative NIS synthetases in the Physalacriaceae were identified as Type A′ NIS synthetases. The identity of the NIS synthetases was based on their clustering with representatives of Type A′ NIS synthetases. The representative synthetases were FslA, LbtA, Rfs, SfaB, and SfaD which synthesize rhizoferrin (Sullivan *et al.* 2006), legiobactin (Allard *et al.* 2006), rhizoferrin (Carroll *et al.* 2017), staphyloferrin A (Cotton *et al.* 2009), and staphylofferin A (Cotton *et al.* 2009), respectively.

Within the Type A′ NIS synthetase group, the NIS synthases of NIS Cluster 1 formed 1 phylogenetic group while NIS synthases in NIS Cluster 2 formed another distinct phylogenetic group. Based on the phylogenetic analysis, the NIS synthetases encoded by the NIS synthase genes in NIS Cluster 1 of all the genomes studied are expected to be more similar to the only characterized fungal NIS synthetase, Rfs, from *R. delemar* (Carroll *et al.* 2017). These results further support the contention that the 2 distinct NIS synthetase gene clusters in the Physalacriaceae may synthesize different siderophores.

#### Characteristics of the putative NIS synthetases are comparable to characterized NIS synthetases of other organisms

IucA_IucC and ferric iron reductase FhuF-like transporter domains at the N- and C-terminals are a conserved domain architecture in several characterized NIS synthetases (Carroll *et al.* 2017). This characteristic feature of known NIS synthetases was also recorded in the putative NIS synthetases encoded by the studied Physalacriaceae excluding that of *A. cepistipes* S262 (Armcep1_13598), which contained a third domain at the C-terminal. The IucA_IucC domain is responsible for biosynthesis of the siderophore whereas Fhuf may be involved in transport (Müller *et al.* 1998; Matzanke *et al.* 2004; Carroll *et al.* 2017; Cain and Smith 2021).

The sizes of the NIS synthetases in the Physalacriaceae determined in this study were generally similar to those of known NIS synthetases. Some of the NIS synthetases were smaller [Oudmuc1_1223755 (S66) and Armtab1_1497269 (S1)] or larger [Armcep1_13598 (S262)] than the known NIS synthetases. The larger size of Armcep1_13598 (S262) is explained by the fact that this gene appeared to be a fused gene consisting of the NIS synthetase gene and the C2 domain-containing protein/MRP-like transporter gene located immediately downstream of the NIS synthetase gene in NIS Cluster 1 of the other genomes studied, excluding that of *O. mucida* S1 (Oudmuc1_1247625). This kind of fusion of 2 genes has been documented in other siderophore BGCs or operons. For instance, Carmichael *et al.* (2019) showed that the homologs of the *IucA* and *IucB* genes in the woodybactin BGC of *Shewanella woodyi* MS32 are fused. In terms of exons, the gene encoding Rfs in *R. delemar* RA 99–880 contains 6 exons (Carroll *et al.* 2017) and was comparable to the number of exons in the *IucA*/*IucC* genes of NIS Cluster 1 in this study. Further research is required to determine the biological implications of the putative NIS synthetase gene modification for *A. cepistipes*.

### Iron-dependent growth and siderophore biosynthesis

Narh Mensah *et al.* (2023) recently reported 1 conserved NRPS-dependent siderophore synthetase gene cluster in *Armillaria* and other members of the Physalacriaceae, and biosynthesis of different types of siderophores by the same strains of *Armillaria* spp. included in the study presented here. In the current study, we identified 2 putative distinct NIS synthetase gene clusters in the genomes studied. Together, these studies suggest that *Armillaria* spp. and other members of the Physalacriaceae have a strong need for iron homeostasis. Thus, research discussed in this section sought to gain insight into the effect of iron on growth and siderophore biosynthesis on species of *Armillaria*.

#### Iron-dependent growth and macromorphology of *Armillaria* species varied on the various media

Growth of various microorganisms has been shown to generally increase with increasing iron concentrations in the growth medium (Manwar *et al.* 2004; Deng *et al.* 2006; Giuliano Garisto Donzelli *et al.* 2015). The effect of iron on both growth rate and extent of growth of the *Armillaria* spp. in the present study, irrespective of the species, is inconsistent with the reported iron-dependent growth of other organisms. This suggests that *Armillaria* spp. may differ in their requirements for iron and that this variation in iron requirement by *Armillaria* spp. is not species-specific.

Various macromorphological characteristics have been reported to be affected by iron concentration in other organisms. This includes the report that morphological characteristics such as number and biomass of microsclerotia as well as melanin production of the fungus, *Nomuraea rileyi*, increase with increasing added iron in the growth medium (Li *et al.* 2016). *Armillaria* spp. in this study generally retained culture macromorphology under the 2 growth conditions, although the observed brownish exudates were more predominant on the iron replete PDPA (PDPA+). The composition of the exudates synthesized by the species included in this study is not known. Knowing the composition would provide a better insight into the effect of iron on biosynthetic properties of species of *Armillaria*.

#### Siderophore biosynthesis occurred irrespective of concentration of added FeCl_3_

In this study, all strains synthesized siderophores with no addition of FeC1_3_ and at 100 µM added FeC1_3_. This concentration is much higher relative to the gram negative bacteria *Pseudomonas putida* strain B 10 (Alexander and Zuberer 1991) and *Pseudomonas aeruginosa* (Manwar *et al.* 2004) that synthesize siderophores at 0–40 µM but not at 50 µM added FeC1_3_·6H_2_O. This finding further supports our assertion that *Armillaria* spp. generally differ in their iron requirements.

Siderophore biosynthesis is usually inversely proportional to the concentration of iron in the growth medium (Fekete *et al.* 1989; Alexander and Zuberer 1991; Manwar *et al.* 2004; Li *et al.* 2016). For instance, the basidiomycetes *Coriolus versicolor* and *Gloeophyllum trabeum* show decreasing siderophore biosynthesis with increasing added FeC1_3_ concentration in vitro at 30 days of incubation (Fekete *et al.* 1989). This trend was not observed in the present study. We propose that the observed FeC1_3_ concentration-independent siderophore production by the studied *Armillaria* spp. may be due to biosynthesis of different siderophores at different concentrations of added FeC1_3_. This phenomenon has been reported in *E. chrysanthemi* (Franza *et al.* 2005).

## Conclusions

In this study, NIS synthetase gene clusters and NIS synthetase genes in genomes of *Armillaria* species were identified, characterized, and compared to other species in the Physalacriaceae. This is the first report of 2 distinct NIS synthetase gene clusters in fungal genomes. Our results suggest that the NIS synthetase gene clusters may synthesize different siderophores and that *Armillaria* species have unique requirements for iron.

Our findings, in concert with Narh Mensah *et al.* (2023), demonstrate that much is still to be discovered about siderophore biosynthesis and utilization in the Basidiomycota. Our BLAST analyses show that there are NIS sequences in other Basidiomycota albeit with low percentage identity. Studies such as gene modification/knock out combined with biochemical characterization (e.g. enzymology, proteomics, and metabolomics) will be needed to fully understand these gene clusters, to characterize the genes and siderophores synthesized, and to determine the biosynthetic models of these BGCs. Other biological studies will be required to determine the role of the siderophores in growth, pathogenicity and/or virulence, and other potential functions of siderophores in *Armillaria* spp. Although, further studies will be needed to confirm the functionality of the identified NIS genes, the knowledge generated from this study, and the suggested studies will unlock avenues for controlling fungal pathogens, such as those belonging to *Armillaria*, and may result in discovery of new biotechnologically useful products.

## Supplementary Material

jkad205_Supplementary_Data

## Data Availability

Publicly available genome and RNA sequences were analyzed in this study. These data can be found at https://mycocosm.jgi.doe.gov/mycocosm/species-tree/tree;05h0Ue?organism=physalacriaceae. Unpublished genome and RNA sequence data obtained from JGI were used with permission from Dr. László G. Nagy. The authors affirm that all data necessary for confirming the conclusions of the article are present within the article, figures, tables, and supporting materials. Supplemental material available at G3 online.
