# Supplementary material for: Two distinct non-ribosomal peptide synthetase-independent siderophore synthetase gene clusters identified in Armillaria and other species in the Physalacriaceae
Source: G3 (Bethesda). 2023 Oct 16;13(12):jkad205. doi: 10.1093/g3journal/jkad205 (PMC10700112; doi:10.1093/g3journal/jkad205)
Supplement: jkad205_Supplementary_Data [file jkad205_supplementary_data.zip › Supplemental_Material_Legends_G3-2023-404446.docx]

# Supplementary Material Legends

Table S1: Source information for genomes analyzed

Table S2: Information about putative proteins coded by genes in NIS Clusters 1 and 2 of *A. borealis*

Table S3: ClusterBlast details of Armbor1 S7 cluster showing 100% gene similarity with NW_006267366 in *Agaricus bisporus* var. *bisporus* H97

Table S4: Information about the amino acid sequences of the NIS synthetases used for the phylogenetic analysis and other characteristics of the putative NIS synthetases of the Physalacriaceae

Figure S1: Workflow for *in silico* analyses of NIS synthetase SMGCs

**File S1:** Compiled tBLASTn results for fungiSMASH-detected NIS clusters

**File S2:** Iron-dependent growth of Armillaria species

**File S3:** Spectrophotometric readings for CAS assa
