# Supplementary material for: Two distinct non-ribosomal peptide synthetase-independent siderophore synthetase gene clusters identified in Armillaria and other species in the Physalacriaceae
Source: G3 (Bethesda). 2023 Oct 16;13(12):jkad205. doi: 10.1093/g3journal/jkad205 (PMC10700112; doi:10.1093/g3journal/jkad205)
Supplement: jkad205_Supplementary_Data [file jkad205_supplementary_data.zip › Table S1.docx]

**Table S1:** Source information for genomes analyzed

| **Species ^a^** | **Genome code ^b^** | **Size (Mb)** | **N50 ^c^** | **GC content (%) ^c^** | **Complete BUSCOs (%) ^d^** | **NCBI accession number or link to genome** | **Reference** |
| --- | --- | --- | --- | --- | --- | --- | --- |
| *Armillaria borealis*  [FPL87.14 v1.0] | Armbor1 | 71.69 | 355,568 | 48.28 | 84.7, 12.9 | https://mycocosm.jgi.doe.gov/Armbor1/Armbor1.home.html | Grigoriev et al. (2014) |
| *Armillaria cepistipes*  [B5] | Armcep1 | 75.50 | 3,291,351 | 47.71 | 97.8, 1.0 | FTRY00000000.1 | Sipos et al. (2017) |
| *Armillaria fumosa*  [CBS 122221 v1.0] | Armfum1 | 55.82 | 3,850,518 | 48.02 | 97.7, 1.1 | https://mycocosm.jgi.doe.gov/Armfum1/Armfum1.home.html | Grigoriev et al. (2014) |
| *Armillaria mellea*  [ELDO17 v1.0] | Armme1 | 70.86 | 2,540,624 | 47.81 | 80.8, 15.6 | https://mycocosm.jgi.doe.gov/Armmel1/Armmel1.home.html | Grigoriev et al. (2014) |
| *Armillaria nabsnona* [CMW6904 v1.0] | Armnabs1 | 62.72 | 2,689,454 | 47.79 | 98.2, 0.7 | https://mycocosm.jgi.doe.gov/Armnab1/Armnab1.home.html | Grigoriev et al. (2014) |
| *Armillaria novae-zelandiae* [2840 v1.0] | Armnov1 | 79.33 | 741,865 | 47.53 | 84.2, 12.6 | https://mycocosm.jgi.doe.gov/Armnov1/Armnov1.home.html | Grigoriev et al. (2014) |
| *Cylindrobasidium torrendii* [FP15055 v1.0] | Cylto1 | 40.60 | 89,590 | 51.92 | 95.0, 1.3 | JYFH00000000.1 | Floudas et al. (2015) |
| *Desarmillaria ectypa*  [FPL83.16 v1.0] | (Des)Armect1 | 74.88 | 4,048,416 | 46.88 | 97.5, 1.0 | https://mycocosm.jgi.doe.gov/Armect1/Armect1.home.html | Grigoriev et al. (2014) |
| *Desarmillaria tabescens* [CCBAS 213 v1.0] | (Des)Armtab1 | 31.57 | 543,822 | 47.89 | 79.2, 16.8 | https://mycocosm.jgi.doe.gov/Armtab1/Armtab1.home.html | Grigoriev et al. (2014) |
| *Guyanagaster necrorhizus* [MCA 3950 v1.0] | Guyne1 | 53.69 | 1,338,049 | 45.5 | 97.8, 0.7 | JAEACO000000000.1 | Koch et al. (2021) |
| *Oudemansiella mucida* (*Mucidula mucida*)  [CBS 558.79 v1.0] | Oudmuc1 | 61.73 | 122,565 | 50.03 | 72.0, 20.0 | JADNYV000000000.1 | Ruiz-Duenas et al. (2021) |

^a^: Presented as species name [Strain or isolate]

^b^: (Des) has been included in the original genome code to indicate that these species belong to the genus *Desarmillaria.*

^c^: Determined using assembled genome sequences with the QUAST tool in Galaxy. The genome assembly mode, and “Fungus use of GeneMark-ES for gene finding, Barrnap for ribosomal RNA genes prediction” were used in QUAST.

^d^: Determined using assembled genome sequences with BUSCO version 5.4.6. Augustus was employed for gene prediction using the agaricales_odb10 lineage dataset (n=3,870). Values are presented as percentage single copy BUSCOs, followed by percentage duplicated BUSCOs.

**References**

Floudas, D., Held, B.W., Riley, R., Nagy, L.G., Koehler, G., Ransdell, A.S., et al. (2015). Evolution of novel wood decay mechanisms in Agaricales revealed by the genome sequences of *Fistulina hepatica* and *Cylindrobasidium torrendii*. *Fungal Genet. Biol.* 76**,** 78-92. doi: 10.1016/j.fgb.2015.02.002

Grigoriev, I.V., Nikitin, R., Haridas, S., Kuo, A., Ohm, R., Otillar, R., et al. (2014). MycoCosm portal: gearing up for 1000 fungal genomes. *Nucleic Acids Res.* 42(D1)**,** D699-D704. doi: 10.1093/nar/gkt1183.

Koch, R.A., Yoon, G.M., Aryal, U.K., Lail, K., Amirebrahimi, M., LaButti, K., et al. (2021). Symbiotic nitrogen fixation in the reproductive structures of a basidiomycete fungus. *Curr. Biol.* 31(17)**,** 3905-3914.e3906. doi: 10.1016/j.cub.2021.06.033.

Ruiz-Duenas, F.J., Barrasa, J.M., Sanchez-Garcia, M., Camarero, S., Miyauchi, S., Serrano, A., et al. (2021). Genomic analysis enlightens Agaricales lifestyle evolution and increasing peroxidase diversity. *Mol. Biol. Evol.* 38(4)**,** 1428-1446. doi: 10.1093/molbev/msaa301.

Sipos, G., Prasanna, A.N., Walter, M.C., O’Connor, E., Bálint, B., Krizsán, K., et al. (2017). Genome expansion and lineage-specific genetic innovations in the forest pathogenic fungi *Armillaria*. *Nat. Ecol. Evol.* 1(12)**,** 1931-1941. doi: 10.1038/s41559-017-0347-8.
