# Supplementary material for: Two distinct non-ribosomal peptide synthetase-independent siderophore synthetase gene clusters identified in Armillaria and other species in the Physalacriaceae
Source: G3 (Bethesda). 2023 Oct 16;13(12):jkad205. doi: 10.1093/g3journal/jkad205 (PMC10700112; doi:10.1093/g3journal/jkad205)
Supplement: jkad205_Supplementary_Data [file jkad205_supplementary_data.zip › Table S4.docx]

**Table S4: Information about the amino acid sequences of the NIS synthetases used for the phylogenetic analysis and other characteristics of the putative NIS synthetases of the Physalacriaceae**

| **NIS type ^a^** | **Organism** | **Accession ^b^** | **Protein** | **No of amino acids** | **Siderophore synthesized** | **Reference(s)** |
| --- | --- | --- | --- | --- | --- | --- |
| Type A | *Bacillus anthracis* | WP_000679659.1 | AsbA | 602 | petrobactin | Lee et al. (2007) |
| Type A | *Dickeya dadantii* 3937 | ADM97831.1 | AcsD | 620 | achromobactin | Schmelz et al. (2009); Glasner et al. (2011) |
| Type A | *Escherichia coli* | AAZ29624.1 | IucA | 575 | aerobactin | de Lorenzo et al. (1986); Sabri et al. (2006); Carroll et al. (2017) |
| Type A | *Sinorhizobium meliloti* | WP_010968203.1 | RhbC | 585 | rhizobactin | Lynch et al. (2001) |
| Type A | *Staphylococcus aureus* | AAP82067.1 | SbnE | 578 | staphyloferrin B | Dale et al. (2004); Cheung et al. (2009) |
| Type A | *Vibrio alginolyticus* | ABM30202.1 | PvsD | 609 | vibrioferrin | Wang et al. (2007); Fujita et al. (2011); Carroll et al. (2017) |
| Type A’ | *Francisella tularensis* | WP_003022888.1 | FslA | 639 | rhizoferrin | Sullivan et al. (2006); Carroll et al. (2017); Li et al. (2021) |
| Type A’ | *Legionella pneumophila* | AAZ39407.1 | LbtA | 580 | legiobactin | Allard et al. (2006) |
| Type A’ | *Rhizopus delemar* RA 99-880 | I1C129.1 | Rfs | 634 | rhizoferrin | Carroll et al., (2017) |
| Type A’ | *Staphylococcus aureus* | WP_072519492.1 | SfaB | 585 | staphyloferrin A | Cotton et al. (2009) |
| Type A’ | *Staphylococcus aureus* | WP_001052566.1 | SfaD | 658 | staphyloferrin A | Cotton et al. (2009) |
| Type B | *Dickeya dadantii* | WP_253940872.1 | AcsA | 647 | achromobactin | Franza et al. (2005) |
| Type B | *Vibrio alginolyticus* | BCB59206.1 | PvsB | 610 | vibrioferrin | Tanabe et al. (2003) |
| Type B | *Staphylococcus argenteus* | WP_000556879.1 | SbnC | 584 | staphyloferrin B | Cheung et al. (2009) |
| Type C | *Bacillus cereus* | WP_011053144.1 | AsbB | 612 | petrobactin | Oves-Costales et al. (2008); Nusca et al. (2012) |
| Type C | *Dickeya chrysanthemi* | WP_040000518.1 | AcsC | 618 | achromobactin | Kadi and Challis, (2009); Carroll et al. (2017) |
| Type C | *Staphylococcus aureus* | AAP82068.1 | SbnF | 579 | staphyloferrin B | Dale et al. (2004); Cheung et al. (2009); |
| Type C’ | *Escherichia coli* | AAS66995.1 | IucC | 580 | aerobactin | de Lorenzo and Neilands (1986); Jørgensen et al. (2019) |
| Type C’ | *Shewanella chilikensis* | BCV36228.1 | PubC | 630 | putrebactin | Kadi et al. (2008) |
| Type C’ | *Streptomyces scabiei* | WP_013003384.1 | DesD | 590 | desferrioxamine | Carroll et al. (2017) |
| C1 | *A. borealis* | Armbor1_1990424 [S7] | NC | 621 | NC | This study |
| C1 | *A. cepistipes* | Armcep1_13598 [S262] | NC | 1132 | NC | This study |
| C1 | *A. fumosa* | Armfum1_800419 [S5] | NC | 613 | NC | This study |
| C1 | *A. mellea* | Armmel1_1031929 [S3] | NC | 613 | NC | This study |
| C1 | *A. nabsnona* | Armnab1_838196 [S9] | NC | 621 | NC | This study |
| C1 | *A. novae-zelandiae* | Armnov1_1416980 [S5] | NC | 614 | NC | This study |
| C1 | *D. ectypa* | Armect1_1382614 [S5] | NC | 621 | NC | This study |
| C1 | *D. tabescens* | Armtab1_1497269 [S1] | NC | 497 | NC | This study |
| C1 | *G. necrorhizus* | Guyne1_882414 [S3] | NC | 621 | NC | This study |
| Oudmuc1 C1 | *O. mucida* | Oudmuc1_1247625 [S1] | IucA/IucC | 602 | NC | This study |
| O1 | *Agaricus bisporus* var. *bisporus* H97 | XP_006459224.1 | NC | 624 | NC | Morin et al. (2012); This study |
| O1 | *Mycena galopus* ATCC 62051 | KAF8207121.1 | IucC | 597 | NC | Miyauchi et al. (2020); This study |
| O1 | *Pleurotus pulmonarius* | KAF4571474.1 | IucA/IucC | 612 | NC | This study |
| C2 | *A. borealis* | Armbor1_2060109 [S46] | IucA/IucC | 555 | NC | This study |
| C2 | *A. cepistipes* | Armcep1_18630 [S270] | IucA/IucC | 543 | NC | This study |
| C2 | *A. fumosa* | Armfum1_1394615 [S10] | IucA/IucC | 555 | NC | This study |
| C2 | *A. mellea* | Armmel1_1066420 [S10] | IucA/IucC | 537 | NC | This study |
| C2 | *A. nabsnona* | Armnab1_70081 [S10] | IucA/IucC | 555 | NC | This study |
| C2 | *D. ectypa* | Armect1_1388155 [S11] | IucA/IucC | 555 | NC | This study |
| C2 | *D. tabescens* | Armtab1_1340322 [S55] | IucA/IucC | 556 | NC | This study |
| C2 | *G. necrorhizus* | Guyne1_910580 [S49] | IucA/IucC | 559 | NC | This study |
| Oudmuc1 C2 | *O. mucida* | Oudmuc1_1223755 [S66] | NC | 483 | NC | This study |
| O2 | *Agaricus bisporus* var. *bisporus* H97 | XP_006459225.1 | NC | 631 | NC | Morin et al. (2012); This study |
| O2 | *Lactarius volemus* | KAH9972089.1 | IucC | 632 | NC | Looney et al. (2022); This study |
| O2 | *Lepista nuda* | KAF9468166.1 | IucA/IucC | 631 | NC | This study |

^a^: Presented as NIS synthetase type for known proteins, cluster in which the proteins are found in the Physalacriaceae (C1, C2, Oudmuc1 C1 and Oudmuc1 C2), and orthologous proteins (O1 and O2) identified by BLASTp searches in the NCBI database. C1=NIS synthetase genes found in NIS Cluster 1, C2=NIS synthetase genes found in NIS Cluster 2, Oudmuc1 C1 and Oudmuc1 C2=NIS synthetase genes of *O. mucida* found in NIS Clusters 1 and 2 respectively, O1 and O2= genes found to be orthologous to the NIS synthetase genes of the Physalacriaceae in NIS Clusters 1 and 2 respectively.

^b^: Accession numbers provided for known proteins or identified orthologs, Genome code followed by ProteinId from JGI and [scaffold number] for the Physalacriaceae

NC = not characterized

**References**

Allard, K.A., Viswanathan, V.K., and Cianciotto, N.P. (2006). lbtA and lbtB are required for production of the *Legionella pneumophila* siderophore legiobactin. *J. Bacteriol.* 188(4)**,** 1351-1363. doi: 10.1128/JB.188.4.1351-1363.2006.

Carroll, C.S., Grieve, C.L., Murugathasan, I., Bennet, A.J., Czekster, C.M., Liu, H., et al. (2017). The rhizoferrin biosynthetic gene in the fungal pathogen *Rhizopus delemar* is a novel member of the NIS gene family. *Int. J. Biochem. Cell Biol.* 89**,** 136-146. doi: 10.1016/j.biocel.2017.06.005.

Cheung, J., Beasley, F.C., Liu, S., Lajoie, G.A., and Heinrichs, D.E. (2009). Molecular characterization of staphyloferrin B biosynthesis in *Staphylococcus aureus*. *Mol. Microbiol.* 74(3)**,** 594-608. doi: 10.1111/j.1365-2958.2009.06880.x.

Cotton, J.L., Tao, J., and Balibar, C.J. (2009). Identification and characterization of the *Staphylococcus aureus* gene cluster coding for staphyloferrin A. *Biochemistry* 48(5)**,** 1025-1035. doi: 10.1021/bi801844c.

Dale, S.E., Doherty-Kirby, A., Lajoie, G., and Heinrichs, D.E. (2004). Role of siderophore biosynthesis in virulence of *Staphylococcus aureus*: identification and characterization of genes involved in production of a siderophore. *Infect. Immun.* 72(1)**,** 29-37. doi: 10.1128/IAI.72.1.29-37.2004.

de Lorenzo, V., Bindereif, A., Paw, B.H., and Neilands, J.B. (1986). Aerobactin biosynthesis and transport genes of plasmid ColV-K30 in *Escherichia coli* K-12. *J. Bacteriol.* 165(2)**,** 570-578. doi: 10.1128/jb.165.2.570-578.1986.

de Lorenzo, V., and Neilands, J.B. (1986). Characterization of *iucA* and *iucC* genes of the aerobactin system of plasmid ColV-K30 in *Escherichia coli*. *J. Bacteriol.* 167(1)**,** 350-355. doi: 10.1128/jb.167.1.350-355.1986.

Franza, T., Mahé, B., and Expert, D. (2005). *Erwinia chrysanthemi* requires a second iron transport route dependent of the siderophore achromobactin for extracellular growth and plant infection. *Mol. Microbiol.* 55(1)**,** 261-275. doi: 10.1111/j.1365-2958.2004.04383.x.

Fujita, M.J., Kimura, N., Sakai, A., Ichikawa, Y., Hanyu, T., and Otsuka, M. (2011). Cloning and heterologous expression of the vibrioferrin biosynthetic gene cluster from a marine metagenomic library. *Biosci. Biotechnol. Biochem.* 75(12)**,** 2283-2287. doi: 10.1271/bbb.110379.

Glasner, J.D., Yang, C.H., Reverchon, S., Hugouvieux-Cotte-Pattat, N., Condemine, G., Bohin, J.P., et al. (2011). Genome sequence of the plant-pathogenic bacterium *Dickeya dadantii* 3937. *J. Bacteriol.* 193(8)**,** 2076-2077. doi: 10.1128/JB.01513-10.

Jørgensen, S.L., Stegger, M., Kudirkiene, E., Lilje, B., Poulsen, L.L., Ronco, T., et al. (2019). Diversity and population overlap between avian and human *Escherichia coli* belonging to sequence type 95. *mSphere* 4(1). doi: 10.1128/mSphere.00333-18.

Kadi, N., Arbache, S., Song, L., Oves-Costales, D., and Challis, G.L. (2008). Identification of a gene cluster that directs putrebactin biosynthesis in *Shewanella* species: PubC catalyzes cyclodimerization of N-hydroxy-N-succinylputrescine. *J. Am. Chem. Soc.* 130(32)**,** 10458-10459. doi: 10.1021/ja8027263.

Kadi, N., and Challis, G.L. (2009). "Siderophore biosynthesis: A substrate specificity assay for nonribosomal peptide synthetase‐independent siderophore synthetases involving trapping of acyl‐adenylate intermediates with hydroxylamine," in *Methods Enzymol.,* ed. D.A. Hopwood. Academic Press), 431-457.

Lee, J.Y., Janes, B.K., Passalacqua, K.D., Pfleger, B.F., Bergman, N.H., Liu, H., et al. (2007). Biosynthetic analysis of the petrobactin siderophore pathway from *Bacillus anthracis*. *J. Bacteriol.* 189(5)**,** 1698-1710. doi: 10.1128/jb.01526-06.

Li, B., Deng, X., Kim, S.H., Buhrow, L., Tomchick, D.R., Phillips, M.A., et al. (2021). Alternative pathways utilize or circumvent putrescine for biosynthesis of putrescine-containing rhizoferrin. *J. Biol. Chem.* 296**,** 100146. doi: 10.1074/jbc.RA120.016738.

Looney, B., Miyauchi, S., Morin, E., Drula, E., Courty, P.E., Kohler, A., et al. (2022). Evolutionary transition to the ectomycorrhizal habit in the genomes of a hyperdiverse lineage of mushroom-forming fungi. *New Phytol.* 233(5)**,** 2294-2309. doi: 10.1111/nph.17892.

Miyauchi, S., Kiss, E., Kuo, A., Drula, E., Kohler, A., Sanchez-Garcia, M., et al. (2020). Large-scale genome sequencing of mycorrhizal fungi provides insights into the early evolution of symbiotic traits. *Nat. Commun.* 11(1)**,** 5125. doi: 10.1038/s41467-020-18795-w.

Morin, E., Kohler, A., Baker, A.R., Foulongne-Oriol, M., Lombard, V., Nagy, L.G., et al. (2012). Genome sequence of the button mushroom *Agaricus bisporus* reveals mechanisms governing adaptation to a humic-rich ecological niche. *Proc. Natl. Acad. Sci. U S A* 109(43)**,** 17501-17506. doi: 10.1073/pnas.1206847109.

Nusca, T.D., Kim, Y., Maltseva, N., Lee, J.Y., Eschenfeldt, W., Stols, L., et al. (2012). Functional and structural analysis of the siderophore synthetase AsbB through reconstitution of the petrobactin biosynthetic pathway from *Bacillus anthracis*. *J. Biol. Chem.* 287(19)**,** 16058-16072. doi: 10.1074/jbc.M112.359349.

Oves-Costales, D., Kadi, N., Fogg, M.J., Song, L., Wilson, K.S., and Challis, G.L. (2008). Petrobactin biosynthesis: AsbB catalyzes condensation of spermidine with N8-citryl-spermidine and its N1-(3,4-dihydroxybenzoyl) derivative. *Chem. Commun. (Camb)* (34)**,** 4034-4036. doi: 10.1039/b809353a.

Sabri, M., Leveille, S., and Dozois, C.M. (2006). A SitABCD homologue from an avian pathogenic *Escherichia coli* strain mediates transport of iron and manganese and resistance to hydrogen peroxide. *Microbiology (Reading)* 152(Pt 3)**,** 745-758. doi: 10.1099/mic.0.28682-0.

Schmelz, S., Kadi, N., McMahon, S.A., Song, L., Oves-Costales, D., Oke, M., et al. (2009). AcsD catalyzes enantioselective citrate desymmetrization in siderophore biosynthesis. *Nat. Chem. Biol.* 5(3)**,** 174-182. doi: 10.1038/nchembio.145.

Sullivan, J.T., Jeffery, E.F., Shannon, J.D., and Ramakrishnan, G. (2006). Characterization of the siderophore of *Francisella tularensis* and role of fslA in siderophore production. *J. Bacteriol.* 188(11)**,** 3785-3795. doi: 10.1128/JB.00027-06.

Tanabe, T., Funahashi, T., Nakao, H., Miyoshi, S., Shinoda, S., and Yamamoto, S. (2003). Identification and characterization of genes required for biosynthesis and transport of the siderophore vibrioferrin in *Vibrio parahaemolyticus*. *J. Bacteriol.* 185(23)**,** 6938-6949. doi: 10.1128/jb.185.23.6938-6949.2003.
