# Supplementary material for: Two distinct non-ribosomal peptide synthetase-independent siderophore synthetase gene clusters identified in Armillaria and other species in the Physalacriaceae
Source: G3 (Bethesda). 2023 Oct 16;13(12):jkad205. doi: 10.1093/g3journal/jkad205 (PMC10700112; doi:10.1093/g3journal/jkad205)
Supplement: jkad205_Supplementary_Data [file jkad205_supplementary_data.zip › Table_S2_G3-2023-404446.docx]

**Table S2:** Information about putative proteins coded by genes in NIS Clusters 1 and 2 of *A. borealis*

| **Protein annotation** | **Protein CDS name** | **Protein size ^a^** | **Pfam accession** | **InterPro entry accession** | **Gene Ontology (GO)** | **Selected** **NCBI ortholog ^b^** | |
| --- | --- | --- | --- | --- | --- | --- | --- |
| **Genes in Armbor1 S7 cluster (NIS Cluster 1)** | | | | | | |  |
| ABC transporter | jgi.p\|Armbor1\|1899603 | 1400 (156.2) | PF00664, PF00005 | IPR003439, IPR011527, IPR003593, IPR027417, IPR036640, IPR017871 | GO:0055085, GO:0005524, GO:0140359, GO:0016887, GO:0016020 | XM_043190246.1 (100, 80.71, 0.0) | |
| Histone acyltransferase | jgi.p\|Armbor1\|1704428 | 1479 (163.6) | PF00628, PF17772, PF01853 | IPR013083, IPR016181, IPR001965, IPR019787, IPR011011, IPR002717, IPR040706, IPR019786, IPR036388 | GO:0006355, GO:0016573, GO:0004402 | XM_037359142.1 (43, 50.23, 0.0) | |
| S-adenosyl-L methionine-dependent methyltransferase | jgi.p\|Armbor1\|1778568 | 469 (52.3) | PF01189 | IPR001678, IPR029063, IPR023267 | GO:0001510, GO:0008168 | XM_043185102.1 (96, 91.17, 0.0) | |
| P-loop containing nucleoside triphosphate protein | jgi.p\|Armbor1\|1990336 | 868 (97.6) | PF13625, PF04851, PF16203 | IPR001650, IPR027417, IPR032830, IPR014001, IPR032438, IPR006935, IPR001161 | GO:0006367, GO:0006289, GO:0005524, GO:0016787, GO:0003677, GO:0003678 | XM_041317970.1 (97, 82.72, 0.0) | |
| Uracil DNA glycosylate-like protein | jgi.p\|Armbor1\|1778607 | 376 (41.3) | PF03167 | IPR018085, IPR002043, IPR005122, IPR036895 | GO:0006281, GO:0006284, GO:0016799, GO:0004844 | XM_043189853.1 (100, 81.12, 0.0) | |
| ABC transporter 1 | jgi.p\|Armbor1\|1778635 | 1409 (157.0) | PF00664, PF00005 | IPR003439, IPR011527, IPR003593, IPR027417, IPR036640, IPR017871 | GO:0055085, GO:0005524, GO:0140359, GO:0016887, GO:0016020 | XM_043180774.1 (99, 87.54, 0.0) | |
| IucA/IucC family domain containing protein (NIS synthetase gene) | jgi.p\|Armbor1\|1990424 | 621 (69.4) | PF04183, PF06276 | IPR037455, IPR007310, IPR022770 | GO:0019290 | XM_046234057.1 (95, 60.70, 0.0) | |
| C2 domain-containing protein / MRP-like transporter | jgi.p\|Armbor1\|1303686 | 506 (54.8) | PF00168 | IPR035892, IPR000008, IPR037791 | N/A | XM_037359091.1 (35, 51.89, 1e-51) | |
| Frag/DRAM/Sfk1 | jgi.p\|Armbor1\|1303804 | 251 (28.4) | PF10277 | IPR019402 | N/A | XM_048024330.1 (98, 52.42, 4e-85) | |
| Cytochrome P450 | jgi.p\|Armbor1\|1732297 | 329 (37.2) | PF00067 | IPR036396, IPR017972, IPR002401, IPR001128 | GO:0016705, GO:0005506, GO:0020037, GO:0004497 | XM_043189314.1 (76, 50.00, 3e-79) | |
| Cytochrome P450 1 | jgi.p\|Armbor1\|1778663 | 502 (56.5) | PF00067 | IPR036396, IPR017972, IPR002401, IPR001128 | GO:0016705, GO:0005506, GO:0020037, GO:0004497 | XM_043189314.1 (93, 75.80, 0.0) | |
| **Genes in Armbor1 S46 Cluster (NIS Cluster 2)** | | | | | | |  |
| Ferric reductase-like transmembrane component domain containing protein | jgi.p\|Armbor1\|996651 | 623 (67.6) | PF01794, PF08022 | IPR013130, IPR017927, IPR039261, IPR017938, IPR013112 | GO:0016491 | XM_046228015.1 (94, 54.58, 0.0) | |
| Aconitate hydratase | jgi.p\|Armbor1\|1827031 | 784 (84.6) | PF00330, PF00694 | IPR015932, IPR015928, IPR015931, IPR001030, IPR036008, IPR000573, IPR018136, IPR006248 | GO:0006099, GO:0051539, GO:0003994 | XM_043181510.1 (100, 96.43, 0.0) | |
| D-lactonohydratase-like protein | jgi.p\|Armbor1\|1907016 | 388 (41.7) | PF08450 | IPR011042, IPR013658 | N/A | XM_043187440.1 (100, 83.13, 0.0) | |
| Delta 9-fatty acid desaturase protein | jgi.p\|Armbor1\|1907015 | 416 (47.0) | PF00487, PF00173 | IPR001199, IPR001522, IPR036400, IPR005804, IPR015876, IPR018506 | GO:0006629, GO:0016717, GO:0020037 | XM_001879546.1 (97, 72.35, 0.0) | |
| Phenylacetyl-CoA ligase | jgi.p\|Armbor1\|1826986 | 578 (63.6) | PF00501, PF13193 | IPR000873, IPR042099, IPR045851, IPR025110 | N/A | XM_043188395.1 (97, 92.53, 0.0) | |
| P-loop containing nucleoside triphosphate hydrolase protein | jgi.p\|Armbor1\|995985 | 611 (68.7) | PF08740, PF00004 | IPR014851, IPR027417, IPR003593, IPR003959, IPR003960 | GO:0016887, GO:0005524 | XM_043177958.1 (91, 73.10, 0.0) | |
| Eukaryotic translation initiation factor 3 subunit 6 | jgi.p\|Armbor1\|2010755 | 606 (69.0) | PF10255 | IPR019382 | GO:0003743, GO:0005852, GO:0005737 | XM_001834986.1 (99, 76.28, 0.0) | |
| Protein kinase-like domain-containing protein | jgi.p\|Armbor1\|1826934 | 508 (57.8) | PF00069, PF00433 | IPR011009, IPR017892, IPR000719, IPR000961, IPR008271, IPR017441 | GO:0006468, GO:0004674, GO:0005524, GO:0004672 | XM_043187439.1 (94, 93.14, 0.0) | |
| Glycoside hydrolase family 95 protein | jgi.p\|Armbor1\|2021490 | 855 (93.2) | PF14498 | IPR008928, IPR012341, IPR027414 | GO:0005975 | XM_043190676.1 (95, 82.91, 0.0) | |
| Ubiquitin-conjugating enzyme/RWD-like protein | jgi.p\|Armbor1\|995512 | 229 (25.6) | PF00179 | IPR016135, IPR000608 | N/A | XM_027759635.1 (80, 64.32, 8e-79) | |
| Endoglucanase V-like protein | jgi.p\|Armbor1\|1892064 | 183 (18.5) | N/A | IPR036908, IPR007112 | N/A | XM_007382807.1 (90, 67.82, 2e-71) | |
| SNARE domain-containing protein | jgi.p\|Armbor1\|995449 | 206 (23.1) | PF05739 | IPR000727 | N/A | XM_037362815.1 (90, 40.74, 2e-25) | |
| IucA/IucC family domain containing protein (NIS synthetase gene) | jgi.p\|Armbor1\|2060109 | 555 (61.1) | PF04183, PF06276 | IPR037455, IPR007310, IPR022770 | GO:0019290 | XM_046226752.1 (77, 39.53, 9e-106) | |
| WH1-domain-containing protein | jgi.p\|Armbor1\|2038045 | 455 (47.3) | PF00786 | IPR036936, IPR003124, IPR000697, IPR011993, IPR000095 | GO:0003779 | XM_050346853.1 (43, 51.60, 1e-60) | |
| Glutamyl-tRNA synthetase | jgi.p\|Armbor1\|1892062 | 682 (76.7) | PF00749, PF03950 | IPR020061, IPR020059, IPR020058, IPR020056, IPR011035, IPR014729, IPR000924, IPR036282, IPR004526, IPR001412 | GO:0006418, GO:0043039, GO:0006412, GO:0006424, GO:0005524, GO:0000166, GO:0004812, GO:0004818, GO:0005737 | XM_027756926.1 (98, 55.45, 0.0) | |
| FAD/NAD-P-binding domain-containing protein | jgi.p\|Armbor1\|1973252 | 429 (46.7) | PF01494 | IPR002938, IPR036188 | GO:0071949 | XM_043189205.1 (99, 76.54, 0.0) | |
| Glycoside hydrolase family 61 protein | jgi.p\|Armbor1\|2085613 | 291 (30.4) | PF03443 | IPR005103 | N/A | XM_043181506.1 (100, 74.91, 1e-156) | |
| Glycoside hydrolase family 61a protein | jgi.p\|Armbor1\|1973221 | 321 (32.9) | PF03443 | IPR005103 | N/A | XM_043181506.1 (98, 56.85, 4e-114) | |
| Cellobiose dehydrogenase | jgi.p\|Armbor1\|1892058 | 715 (76.5) | PF16010, PF13450, PF00732, PF05199 | IPR015920, IPR000172, IPR036188, IPR007867 | GO:0050660, GO:0016614 | XM_043181505.1 (98, 77.54, 0.0) | |
| Mitochondrial carrier protein RIM2 | jgi.p\|Armbor1\|1892056 | 317 (34.0) | PF00153, PF00153, PF00153 | IPR023395, IPR018108, IPR002067 | GO:0055085 | XM_043183799.1 (96, 90.23, 0.0) | |
| HSF type DNA-binding-domain containing protein | jgi.p\|Armbor1\|1714821 | 672 (74.5) | PF00447, PF00072 | IPR011006, IPR000232, IPR036388, IPR027725, IPR001789 | GO:0006355, GO:0000160, GO:0003700, GO:0043565 | XM_048025672.1 (75, 68.08, 3e-105) | |

Information is presented in the order of gene appearance from left to right on the respective synteny maps (Figure 1 and Figure 2). Information about hypothetical/uncharacterized proteins are excluded from the table. All information excluding NCBI information was obtained from InterPro. The genes in the clusters reported for Armbor1 are representative of the genes in the clusters of the other genomes studied. N/A = none available.

^a^: Presented as number of amino acids (weight in kDa). Protein weight of protein coding sequences (CDS) of the respective genes obtained from annotated genomes in CLC Main Workbench was estimated to one decimal point using the ‘Create Protein Report’ tool in CLC Main Workbench.

^b^: NCBI ortholog information was obtained from tBLASTn searches using the CDSs of the respective genes. Data is presented as Accession (Query Cover, Percentage Identity, Expected value). Query Cover and Percentage Identity are in %.
