# Supplementary material for: Two distinct non-ribosomal peptide synthetase-independent siderophore synthetase gene clusters identified in Armillaria and other species in the Physalacriaceae
Source: G3 (Bethesda). 2023 Oct 16;13(12):jkad205. doi: 10.1093/g3journal/jkad205 (PMC10700112; doi:10.1093/g3journal/jkad205)
Supplement: jkad205_Supplementary_Data [file jkad205_supplementary_data.zip › Table_S3_G3-2023-404446.docx]

**Table S3:** ClusterBlast details of Armbor1 S7 cluster showing 100% gene similarity with NW_006267366 in *Agaricus bisporus* var. *bisporus* H97

| **Armbor1 S7 CASSIS cluster information (21,784 - 33,963 nt.)** | | **BLASTP Hit NW_006267366 (1141250-1156888) genes** | | | | **BLASTP Ortholog** | | | |
| --- | --- | --- | --- | --- | --- | --- | --- | --- | --- |
| **Putative protein name** | **Location** | **Protein Id** | **Percentage coverage (Percentage identity)** | **E-value** | **NCBI Conserved Domain hits** | **Protein name [Organism]** | **Protein ID** | **Percentage coverage (Percentage identity)** | **E-value** |
| IucA/IucC family domain containing protein | 26783 - 28963 | XP_006459225.1 | 99 (44) | 2.1e-146 | COG4264,  Pfam04183, NF033586 | siderophore [*Pleurotus pulmonarius*] | KAF4571475.1 | 99 (68.42) | 0.0 |
|  |  | XP_006459224.1 | 99 (57) | 1.8e-198 | COG4264, pfam04183, NF033583 | IucC family-domain-containing protein [*Lepista nuda*] | KAF9468165.1 | 98 (60.29) | 0.0 |
| C2 domain-containing protein / MRP-like transporter | 29243 - 30866 | XP_006459223.1 | 50 (50) | 1.3e-56 | Cd08681, pfam00168, smart00239, PHA03247, COG5038 | C2 domain-containing protein [*Lepista nuda*] | KAF9468164.1 | 36 (65.43) | 7e-74 |
| Frag/DRAM/Sfk1 | 31062 - 32138 | XP_006459222.1 | 92 (63) | 1.7e-78 | Pfam10277, COG0474 | Protein sfk1 [*Leucoagaricus* sp. SymC.cos] | KXN81326.1 | 89 (78.60) | 2e-107 |
