# Supplementary figures and images for: Two distinct non-ribosomal peptide synthetase-independent siderophore synthetase gene clusters identified in Armillaria and other species in the Physalacriaceae
Source: G3 (Bethesda). 2023 Oct 16;13(12):jkad205. doi: 10.1093/g3journal/jkad205 (PMC10700112; doi:10.1093/g3journal/jkad205)

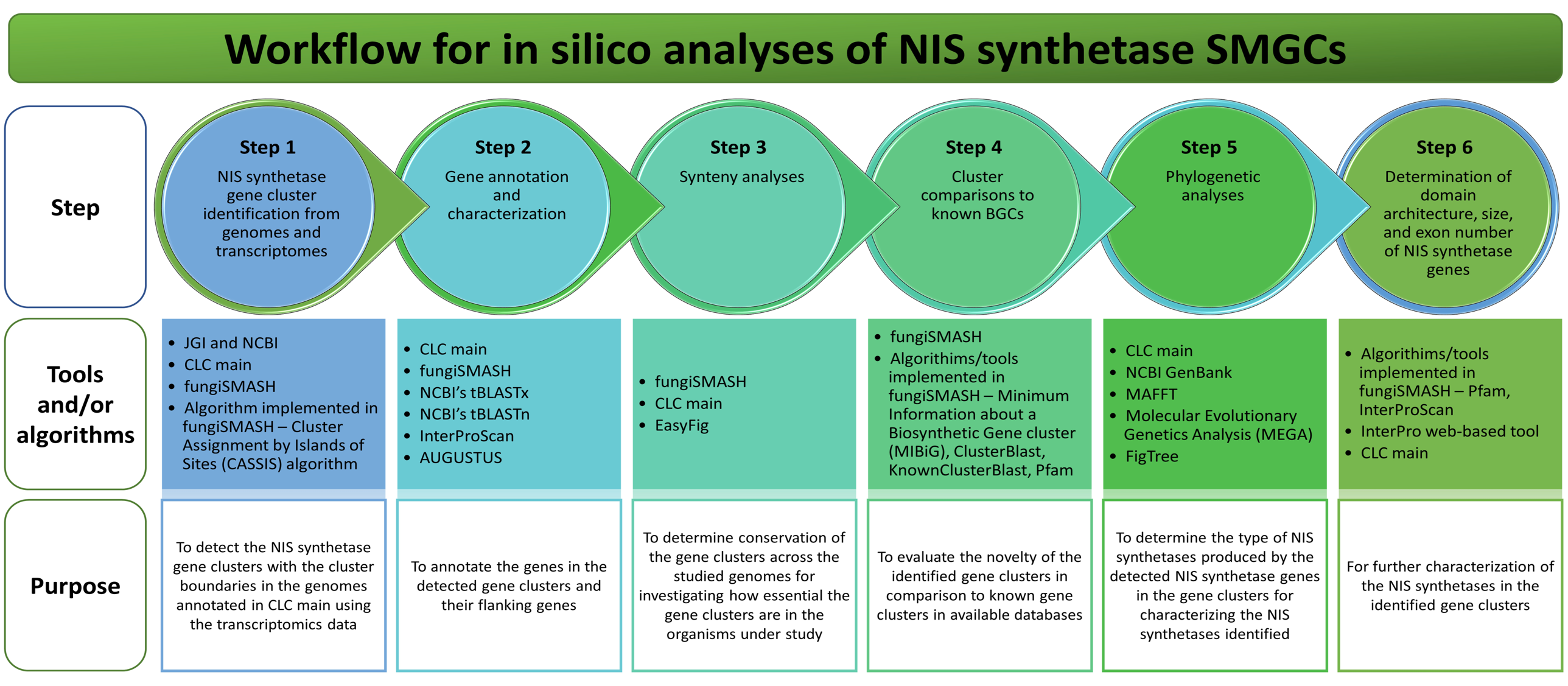

Supplement: jkad205_Supplementary_Data [file jkad205_supplementary_data.zip › Figure_S1_G3-2023-404446.png]
